# Supplementary figures and images for: The TFIIH complex is required to establish and maintain mitotic chromosome structure
Source: eLife. 2022 Mar 16;11:e75475. doi: 10.7554/eLife.75475 (PMC8956287; doi:10.7554/eLife.75475)

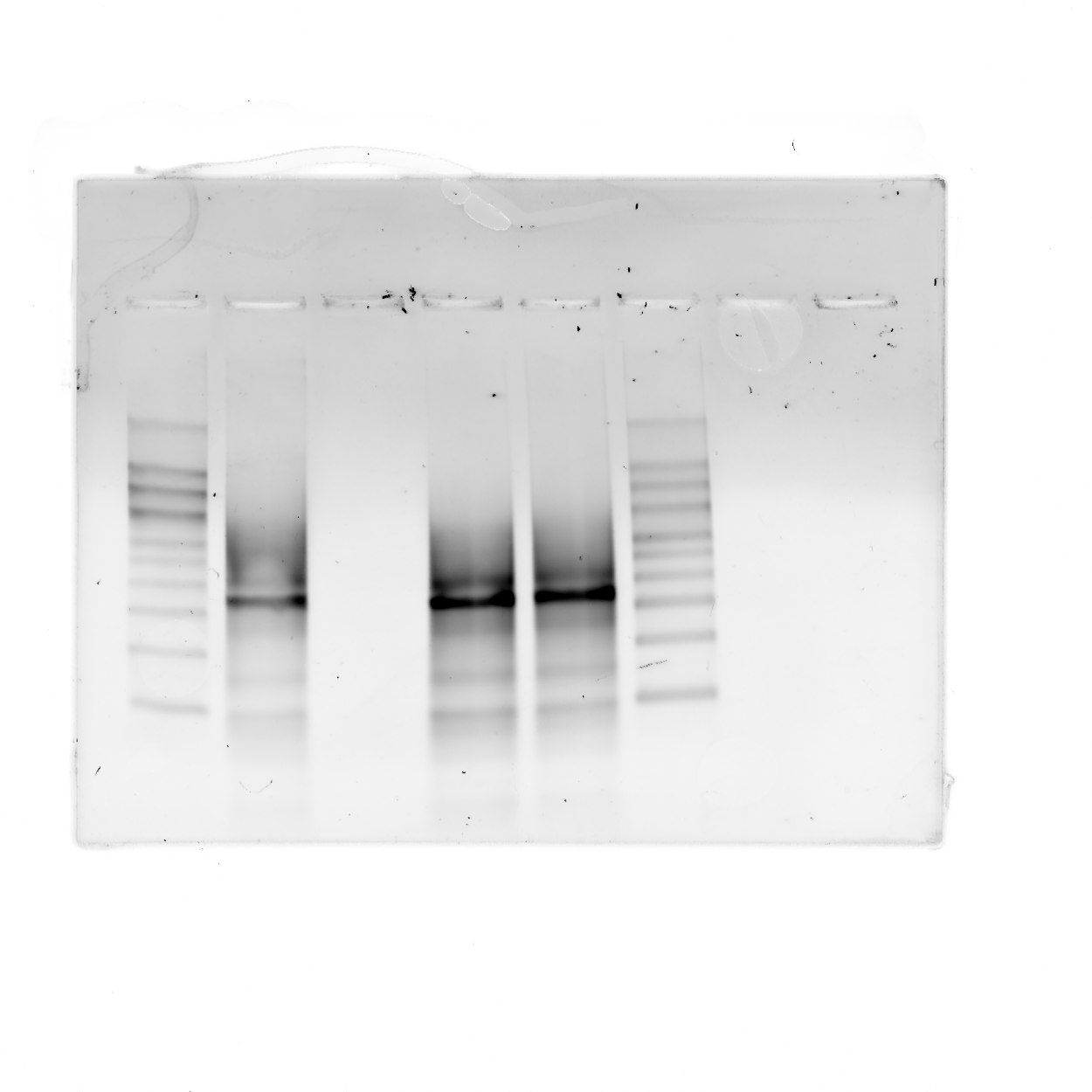

Supplement: Figure 1—figure supplement 1—source data 1. [file elife-75475-fig1-figsupp1-data1.zip › Figure 1 - figure supplement 1B 20201020 Exp 55 RNase AH-[SYBR Safe].tif]
