# Supplementary material for: The TFIIH complex is required to establish and maintain mitotic chromosome structure
Source: eLife. 2022 Mar 16;11:e75475. doi: 10.7554/eLife.75475 (PMC8956287; doi:10.7554/eLife.75475)
Supplement: Figure 5—figure supplement 1—source data 1. [file elife-75475-fig5-figsupp1-data1.zip › 10112021RAeLife75475R2 - Copy.docx]

The TFIIH Complex is Required to Establish and Maintain Mitotic Chromosome Structure

Julian Haase^1^, Richard Chen^1,2,*^, Wesley M. Parker^1*^, Mary Kate Bonner^1^, Lisa M. Miller Jenkins^3^ & Alexander E. Kelly^1,†^

^1^Laboratory of Biochemistry & Molecular Biology, National Cancer Institute, NIH, Bethesda, Maryland 20892

^2^Current address: Graduate School of Biomedical Sciences, UT Southwestern Medical Center, Dallas, Texas 75390

^3^Laboratory of Cell Biology, National Cancer Institute, NIH, Bethesda, Maryland 20892

**Abstract**

Condensins compact chromosomes to promote their equal segregation during mitosis, but the mechanism of condensin engagement with and action on chromatin is incompletely understood. Here, we show that the general transcription factor TFIIH complex is continuously required to establish and maintain a compacted chromosome structure in transcriptionally silent *Xenopus* egg extracts. Inhibiting the DNA-dependent ATPase activity of the TFIIH complex subunit XPB rapidly and reversibly induces a complete loss of chromosome structure and prevents the enrichment of condensins I and II, but not topoisomerase II, on chromatin. In addition, inhibiting TFIIH prevents condensation of both mouse and *Xenopus* nuclei in *Xenopus* egg extracts, which suggests an evolutionarily conserved mechanism of TFIIH action. Reducing nucleosome density through partial histone depletion restores chromosome structure and condensin enrichment in the absence of TFIIH activity. We propose that the TFIIH complex promotes mitotic chromosome condensation by dynamically altering the chromatin environment to facilitate condensin loading and condensin-dependent loop extrusion.

**Introduction**

Entry into mitosis triggers dramatic structural changes in chromosomes that are required for their equal segregation (Zhou and Heald, 2020). A process known as chromosome condensation compacts sister chromatids into rod-like structures to promote individualization and prevent DNA damage during division. Sister chromatids form linear rod-like structures and separate from each other during prophase, and then shorten and thicken during prometaphase, ultimately forming fully condensed metaphase chromosomes. Electron microscope analyses of isolated mitotic chromosomes suggested that DNA is organized into loops around a protein scaffold that is concentrated along the central axis of each chromatid (Earnshaw and Laemmli, 1983; Paulson and Laemmli, 1977; Paulson et al., 2021). In agreement with this, recent studies using chromosome conformation capture and modeling indicate that a sequential helical arrangement of loops is formed around a central axis (Naumova et al., 2013; Gibcus et al., 2018; Elbatsh et al., 2019), although alternative models of loop arrangement have been proposed (Chu et al., 2020). How these elements of chromosome structure are established and maintained during chromosome segregation remains a major question in biology.

The evolutionary conserved five-subunit condensin complex is an ATPase that serves as the primary driver of mitotic chromosome condensation (Kinoshita and Hirano, 2017). Many eukaryotes express two different forms of the condensin complex, condensin I and II (Hirano et al., 1997; Ono et al., 2003). Condensin I and II share two structural maintenance of chromosomes (SMC) subunits (SMC2 and SMC4) that form a V-shaped dimer with two ATP-binding head domains, but have distinct regulatory subunits (CAP-H/H2, CAP-D2/D3, and CAP-G/G2). Condensin I is only loaded onto chromosomes during mitosis, whereas condensin II is present on chromatin throughout the cell cycle (Hirota et al., 2004; Ono et al., 2004; Walther et al., 2018). During mitosis, both condensin complexes are concentrated along the central axis of individual chromatids, but have been demonstrated to have distinct roles in maintaining the lateral and axial compaction of chromatids (Green et al., 2012; Samejima et al., 2012; Bakhrebah et al., 2015). A recent study in chicken DT40 cells proposed that condensin II initially forms large DNA loops, which are then subdivided into smaller loops by condensin I (Gibcus et al., 2018). However, in *Xenopus* egg extracts, condensin II is not essential for the formation of condensed chromatids but remains important for optimal chromosome morphology and proper centromere function (Shintomi and Hirano, 2011; Bernad et al., 2011). Topoisomerase IIα (topo II) is an ATP-dependent DNA strand-passing enzyme that is required to individualize and resolve chromatids that is also involved in chromosome condensation (Kinoshita and Hirano, 2017). Like the condensins, topo II is localized to the mitotic chromatid axis, and current models suggest it acts to control the axial compaction of chromosomes through the regulation of condensin-mediated loops (Nielsen et al., 2020; Shintomi and Hirano, 2021; Paulson et al., 2021).

Multiple factors promote the association of condensin with chromatin (Robellet et al., 2017), including but not limited to, histones (Ball et al., 2002; Liu et al., 2010; Tada et al., 2011), nucleosome-free regions (Piazza et al., 2014; Toselli-Mollereau et al., 2016), single-stranded DNA (Sakai et al., 2003; Sutani et al., 2015), and supercoiling (Kimura et al., 1998; Kim et al., 2021). Tuning condensin levels on chromatin is important for proper chromosome condensation, as prior studies have shown that altering condensin levels leads to gross changes in the axial and lateral compaction of chromosomes, as well as the alteration of the size of extruded DNA loops (Shintomi and Hirano, 2011; Thadani et al., 2018; Elbatsh et al., 2019; Choppakatla et al., 2021; Goloborodko et al., 2016a; Fitz-James et al., 2020; Goloborodko et al., 2016b). However, how condensin levels and activity on chromosomes are regulated remains an outstanding question. One conserved feature of condensins across multiple eukaryotes is their enrichment at sites of RNA polymerase II (Pol II)-dependent transcription (Bernard and Vanoosthuyse, 2015; D'Ambrosio et al., 2008; Schmidt et al., 2009; Nakazawa et al., 2015; Sutani et al., 2015; Kim et al., 2016; Kranz et al., 2013; Kim et al., 2013; Dowen et al., 2013). This suggests a potentially conserved role for transcription or the transcriptional machinery in regulating condensin loading and function during mitosis. However, the role of transcription in mitotic chromosome condensation is not well understood (Robellet et al., 2017). Here, we show that the TFIIH complex, part of the transcriptional pre-initiation complex, is continuously required for chromosome condensation by promoting condensin loading and function on chromatin.

**Results**

**The TFIIH complex is required for chromosome condensation in *Xenopus* egg extracts**

To examine possible relationships between transcription-associated activities and mitotic chromosome condensation, we used cell-free *Xenopus* M phase egg extracts, a system in which condensin function is well characterized and in which zygotic transcription is inactive (Kinoshita et al., 2015; Hirano and Mitchison, 1994; Barrows and Long, 2019; Chen et al., 2019; Amodeo et al., 2015; Jukam et al., 2017). As previously demonstrated (Hirano and Mitchison, 1993; Shintomi et al., 2015), sperm nuclei incubated with a high-speed supernatant (HSS) of egg extracts first swell during the exchange of protamines for maternal histones, assemble into a diffuse “cloud” of chromatin, and gradually form clusters of condensed single-chromatid structures (Figure 1A). As expected, depletion of condensins I and II with anti-CAP-E antibodies caused a severe loss of condensation (Figure 1B and Figure 1–figure supplement 1A), and this effect could be quantified using a previously described “condensation parameter” (Figure 1C) (Maddox et al., 2006). We then asked if various inhibitors of the Pol II-dependent transcriptional machinery affect chromosome condensation in egg extracts when added at the beginning stages of the condensation process (t = 25’), after protamine exchange and “cloud” formation (Figure 1A) (Shintomi et al., 2015). Inhibitors of RNA polymerase II elongation, such as α-amanitin and 5,6-dichloro-1-beta-D-ribofuranosylbenzimidazole (DRB) had no effect on chromosome condensation (Figure 1B and 1C), but induced the loss of the actively elongating (S2ph) form of RNA polymerase II at centromeres as expected (Figure 1–figure supplement 2A and B) (Grenfell et al., 2016; Blower, 2016; Perea-Resa et al., 2020). In addition, condensation was not altered by pretreatment of extracts with RNase A to remove endogenous RNAs (Figure 1–figure supplement 1B and C and Figure 1–figure supplement 3A) or by pretreatment with RNase H to remove RNA-DNA hybrids (Figure 1–figure supplement 1C and Figure 1–figure supplement 3A) (Thakur and Henikoff, 2020). These findings, combined with the fact that very little transcription occurs in *Xenopus* embryos before the maternal-to-zygotic transition (Jukam et al., 2017; Chen et al., 2019), indicates that active transcription is not required for chromosome condensation.

In contrast, treatment of extracts with the transcription initiation inhibitor triptolide completely blocked chromosome condensation, resulting in decondensed chromatin masses similar to structures observed upon condensin depletion (Figure 1B and C) (Ono et al., 2003; Shintomi and Hirano, 2011). Triptolide is an inhibitor of the multi-subunit TFIIH complex (Figure 1D), which as part of the pre-initiation complex is required for Pol II-dependent transcription initiation (Titov et al., 2011; Compe and Egly, 2012). Triptolide covalently binds to the XPB subunit of TFIIH and blocks its DNA-dependent ATPase (Titov et al., 2011). The ATPase activity of XPB is required for the DNA translocase function of TFIIH, which in collaboration with the pre-initiation complex, is essential to twist and open DNA to allow RNA polymerase II to productively engage the template strand to initiate transcription (Fishburn et al., 2015; Tomko et al., 2017; Kim et al., 2000; Grünberg et al., 2012; Nogales and Greber, 2019; Schilbach et al., 2021). In agreement with these described functions of TFIIH, triptolide prevented the accumulation of both paused (S5ph) and actively elongating (S2ph) forms of Pol II at centromeres (Figure 1–figure supplement 2A and B).

To rule out off-target effects of triptolide, we also examined chromosome condensation in extracts depleted of XPB (>95%) using anti-XPB antibodies (Figure 1E and Figure 1–figure supplement 1D). XPB depletion also depleted the TFIIH subunit p62 (Figure 1E), and mass spectrometry analysis of proteins co-depleted with XPB identified strong enrichment of other core TFIIH complex members (Figure 1F). As shown in Figure 1G, XPB depletion completely abrogated chromosome condensation (Figure 1–figure supplement 3B), thus confirming that XPB is the physiological target of triptolide and demonstrating an unprecedented role for the TFIIH complex in chromosome condensation.

The TFIIH complex is involved in processes other than transcription initiation that could also potentially affect chromosome condensation, including the nucleotide excision repair (NER) pathway (Compe and Egly, 2012). Consistent with a previous report (Ito et al., 2007), we identified the NER endonuclease XPG as a strong interactor with the TFIIH complex (Figure 1F). During NER, XPG and ERCC1-XPF, another NER endonuclease, act together to resect DNA lesions after the XPB and XPD subunits of TFIIH have unwound DNA around the lesion (Kolesnikova et al., 2019; Compe and Egly, 2012). To address whether the NER pathway is required for condensation, we immunodepleted ERCC1 from egg extracts, which prevents NER (Klein Douwel et al., 2017; 2014). Depletion of the ERCC1/XPF complex did not perturb chromosome condensation (Figure 1H and 1I, Figure 1–figure supplement 1E, and Figure 1–figure supplement 3C), indicating that the TFIIH complex does not act through the NER pathway to promote chromosome condensation.

TFIIH also associates with the three protein CAK complex (CDK-Activating Kinase) (Figure 1F), which contains the kinase CDK7. CDK7 directly phosphorylates the Pol II C-terminus at Ser5 (Pol II S5ph), and is required for the release of Pol II from the pre-initiation complex to promote transcriptional pausing and other transcriptional events (Rimel and Taatjes, 2018; Glover-Cutter et al., 2009; Akhtar et al., 2009; Ebmeier et al., 2017). As expected, inhibition of CDK7 with THZ1 (Kwiatkowski et al., 2014) induced the loss of the paused and elongating forms of Pol II from centromeres (Figure 1–figure supplement 2A and B). However, THZ1 treatment had no effect on condensation (Figure 1–figure supplement 1F and Figure 1–figure supplement 3D). Furthermore, inhibition of CDK7 did not rescue the condensation defects observed in triptolide-treated extracts, ruling out abnormal upregulation of CDK7 activity upon inhibition of XPB (Figure 1–figure supplement 1F and Figure 1–figure supplement 3D). CDK7, as part of the labile CAK complex, is also required for the initial activation of CDK1 to promote entry into mitosis (Larochelle et al., 2007), and thus we tested the possibility that triptolide exerts its effects on condensation through down-regulation of CDK1 activity. However, triptolide did not affect CDK1 activity or M phase maintenance in HSS extracts, as indicated by unaltered CDK1 activation loop phosphorylation (T161ph) and histone H3T3ph levels ((Kelly et al., 2010); Figure 1–figure supplement 1G). Thus, the ATPase activity of the TFIIH complex is required for condensation in a manner that is independent of TFIIH activities in NER, active transcription, and the cell cycle.

**The TFIIH complex is required for the dynamic maintenance of mitotic chromosome structure**

Chromosome condensation must be actively maintained during mitosis (Kinoshita et al., 2015), but prior work has suggested that condensation establishment and maintenance may involve distinct mechanisms (Nielsen et al., 2020; Hirano and Mitchison, 1993). To ask if the TFIIH complex is required for the maintenance of chromosome condensation as well as its establishment, we added triptolide at various timepoints during the condensation process: at 25 min after addition of sperm to extracts, at condensation initiation; at 60 min, when chromosomes were partially condensed; and at 180 min, when chromosomes were in a steady state of full condensation (Figure 2A). In DMSO-treated controls, condensation increased steadily during these times until reaching a plateau at 180 min (Figure 2A, and Figure 2–figure supplement 1A). However, addition of triptolide at any of these times during the condensation process, including at 180 min, induced the rapid (less than five minutes) loss of discernable individual chromatids and a diffuse DNA staining pattern, similar to the structures observed upon condensin depletion (Figure 1B), that persisted throughout the rest of the timecourse (Figure 2A, and Figure 2–figure supplement 1A). Thus, TFIIH activity is required for maintaining chromosome condensation as well as its establishment.

To test if the triptolide-induced loss of mitotic chromosome structure is reversible, we allowed chromosomes to completely condense for 180 minutes, treated with triptolide for thirty minutes to induce loss of chromosome structure, and then diluted ten-fold with extracts containing triptolide or DMSO (Figure 2B). Dilution with DMSO-treated extracts resulted in the rapid regain (within 5 min) of chromatid structure, while chromosomes remained uncondensed upon dilution with triptolide-treated extract (Figure 2B and 2C, Figure 2–figure supplement 2A and 2B). These results demonstrate that the effects of TFIIH inhibition on chromosome condensation can be reversed rapidly (Figure 2D), suggesting that the TFIIH complex acts continually in dynamic processes required for condensation.

**TFIIH is required for the enrichment of condensins on mitotic chromatin**

The proper assembly of mitotic chromosomes in egg extracts requires the activities of condensins I and II and Topoisomerase IIα (topo II), which localize along condensed chromatids on a central axis (Samejima et al., 2012; Ono et al., 2003; Hirano and Mitchison, 1993; Shintomi et al., 2015; Earnshaw and Heck, 1985). To test the hypothesis that TFIIH is involved in this localization, we used immunofluorescence to measure levels of XPB, condensin I/II, and topo II on chromatin in the presence or absence of triptolide (added at t = 25’, imaged at t = 180’). In control extracts, the XPB was present in a diffuse pattern over the chromatid mass (Figure 3A), with discrete puncta of XPB visible along the entire length of isolated chromatids (Figure 3A (inset) and Figure 3–figure supplement 1A). Condensins I and II and topo II were each present on the central axis of each chromatid, as previously reported ((Ono et al., 2003; Maeshima and Laemmli, 2003); Figure 3A). Triptolide treatment severely reduced XPB levels on chromatid clusters (Figure 3B; 21% of control), indicating that inhibition of the XPB ATPase affects its association with chromatin. Strikingly, triptolide also caused significant reductions in the levels of CAP-E (37% of control) and CAP-C (31% of control), which are subunits of both condensin I and II, and also reduced levels of the condensin II-specific subunit CAP-D3 (Figure 3B; 25% of control). The condensin that remained on chromosomes was present in a diffuse pattern with no defined axis (Figure 3A). Since the amount of condensin I in extracts is three- to five-fold higher than that of condensin II, and since condensin I is the major driver of chromosome condensation in egg extracts (Ono et al., 2003; Shintomi and Hirano, 2011), we conclude that triptolide prevents the proper loading and enrichment of both condensin I and condensin II. Conversely, triptolide had no significant effect on topo II levels (Figures 3A and 3B; 93% of control), although topo II was no longer concentrated on a central axis. A similar effect on topo II localization is seen upon condensin depletion from egg extracts (Shintomi et al., 2017). Similarly, SUPT16H, a member of the FACT complex implicated in both transcription (Zhou et al., 2020) and chromosome condensation (Shintomi et al., 2015), was present at similar levels in the presence or absence of triptolide (Figure 3A and 3B; 113% of control). CAP-C levels also decreased significantly (61% of control) when fully condensed chromosomes were treated with triptolide at t=180’ for thirty-five minutes (Figure 2–figure supplement 2C). These results demonstrate that the TFIIH complex is required for the enrichment of condensin I/II on chromosomes during condensation establishment and maintenance. They also suggest that the TFIIH complex specifically regulates condensins and not topo II, since TFIIH inhibition alters condensin but not topo II levels on chromatin (Figure 3B) and causes defects in condensation that are similar to condensin depletion but not topo II inhibition (Ono et al., 2003; Shintomi et al., 2017; Hirano and Mitchison, 1993).

In reciprocal experiments, we found that XPB localization to chromatin was maintained following the depletion of condensins (Figure 3–figure supplement 1A). Since both TFIIH and condensin levels on chromatin decreased in response to triptolide (Figure 3B), we hypothesized that TFIIH might promote recruitment of condensin complexes to chromosomes through physical interaction. We found that the condensin II subunit CAP-D3 co-immunoprecipitated with XPB from high-speed supernatants of egg extracts (Figure 3–figure supplement 1B). However, XPB was not co-immunoprecipitated with anti-CAP-E antibodies, even though we observed robust interactions with other condensin I and II subunits including CAP-D3 (Figure 3–figure supplement 1C). These results suggest that any interactions between TFIIH and condensins are weak, and not necessarily direct. Furthermore, TFIIH only partially co-localized with condensins on single chromatids, and the TFIIH complex did not demonstrate the stereotypical axial staining pattern observed for condensins, which argues against a scaffolding role for TFIIH (Figure 3–figure supplement 1D). Altogether, these results show that the TFIIH complex acts upstream of condensin enrichment and weakly associates with the CAP-D3 subunit of condensin II, but indicate that the main role of TFIIH in condensin enrichment is not through direct recruitment.

**TFIIH inhibition perturbs chromosome condensation before condensin and TFIIH are lost from chromosomes**

To further investigate how TFIIH promotes condensation and the enrichment of condensins on chromatin, we determined the kinetics of condensin enrichment with and without TFIIH inhibition. Specifically, we added triptolide twenty-five minutes after addition of sperm nuclei to egg extracts and monitored chromatin morphology and condensin levels on chromatin over time (Figure 4A). As expected, the addition of triptolide led to rapid decondensation (Figures 2A, 4B, and Figure 4–figure supplement 1A), and an amorphous cloud of chromatin without discernable chromatids was seen within five minutes of drug addition. DMSO treated extracts progressed in a stereotypical fashion, with increasing condensation over time (Figure 4B and Figure 4–figure supplement 1A). In contrast, total condensin levels, as monitored by CAP-E staining, increased for the first 10 minutes after triptolide addition, remaining similar to controls within the first twenty minutes before decreasing to 35% of control over the next 135 minutes (Figures 4B and 4C, and Figure 4–figure supplement 2A). Similarly, condensin II and XPB remained at control levels for 10 min after triptolide addition before decreasing relative to controls (Figure 4–figure supplement 2B and C). These results indicate that TFIIH plays an immediate role in promoting condensation, and is also required for the ongoing enrichment of condensins on chromosomes that occurs throughout the condensation process.

**Artificial formation of nucleosome-free regions bypasses the requirement for TFIIH in chromosome condensation**

Several lines of evidence suggest that nucleosomes are a barrier to condensin loading and function on chromosomes. In *Xenopus* egg extracts, condensins display a strong preference for binding to DNA over nucleosomes (Zierhut et al., 2014; Shintomi et al., 2017; Choppakatla et al., 2021). The creation of nucleosome-free regions has been proposed to be important for chromosome condensation in fission yeast (Toselli-Mollereau et al., 2016), and double-stranded DNA has been shown to preferentially activate the ATPase activity of budding yeast condensin (Piazza et al., 2014). The TFIIH complex, which acts as a DNA translocase and facilitates changes to chromatin, might promote condensin binding and condensation by altering the chromatin environment to allow the exposure of DNA. To test this suggestion, we artificially introduced nucleosome-free regions on chromosomes by using anti-H4K12ac antibodies to partially deplete histones H3 and H4 from egg extracts (Zierhut et al., 2014) and examined the effects of triptolide on chromosome condensation. Since *Xenopus* sperm retain paternally derived histones H3 and H4 on chromatin (Shintomi et al., 2015), we used mouse sperm, which have previously been shown to condense in a condensin-dependent manner in *Xenopus* egg extracts (Shintomi et al., 2017) but which contain very low levels of histones H3 and H4 (Brykczynska et al., 2010). We found that triptolide inhibits the condensation of mouse sperm nuclei incubated in *Xenopus* egg extracts (Figures 5A, 5B, Figure 5–figure supplement 1A, and Figure 5–figure supplement 2A), indicating that TFIIH activity is required for condensation regardless of chromatin source. Control depletions with IgG did not perturb condensation, nor did it alter the inhibitory effects of triptolide (Figure 5A and B, Figure 5–figure supplement 1B, and Figure 5–figure supplement 2A). Remarkably, depletion of total histone H3 levels in extracts by only 24%, which reduced histone H3 levels on chromosomes by 38% (Figures 5B, 5C, and Figure 5–figure supplement 1B), rescued chromosome condensation in the presence of triptolide (Figure 5A and Figure 5–figure supplement 2A). Importantly, chromosomes were resistant to decondensation ten minutes after TFIIH inhibition, suggesting a direct effect of histone H3/H4 depletion (Figure 5A, and Figure 5–figure supplement 2A). Triptolide treatment caused substantial reductions in both XPB and condensin I/II subunit CAP-E on mouse chromosomes (respectively to 23% and 40% of control levels, Figures 5B and 5C), as it did with *Xenopus* chromosomes (Figures 3A and 3B). However, partial depletion of histones H3 and H4 resulted in a substantial increase in CAP-E levels (to 79% of control levels), and CAP-E labeled the chromatid axis as in controls (Figure 5B and 5C). In contrast, histone H3/H4 depletion did not suppress the triptolide-induced loss of XPB levels from chromosomes (Figures 5B and 5C and Figure 5–figure supplement 1B), indicating that histone depletion does not affect the inhibition of TFIIH by triptolide. The finding that TFIIH function in condensation can be bypassed by partial histone depletion strongly suggests that the main role of TFIIH in condensation is to alter the chromatin environment, possibly by regulating nucleosome composition and/or inhibitory proteins, which in turn promotes condensin function and enrichment on chromatin.

**Discussion**

**Roles of the TFIIH Complex in Chromosome Condensation**

The prevailing model of chromosome condensation posits that condensins extrude loops of chromatin in an ATP-dependent manner to promote the formation of multiple intra-chromatid contacts (Davidson and Peters, 2021). Nucleosomes may form a barrier to both condensin loading and loop extrusion, since condensins bind more strongly to nucleosome-free DNA than to nucleosomes (Zierhut et al., 2014; Shintomi et al., 2017; Toselli-Mollereau et al., 2016; Choppakatla et al., 2021) and the binding to free DNA activates condensin ATPase activity (Piazza et al., 2014). *In vitro* experiments have reported that condensin can extrude loops of chromatinized DNA, but nucleosomes were present in these studies at low densities sufficient to expose naked DNA and permit condensin loading (Kong et al., 2020). Because mitotic chromatin *in vivo* is more compact and nucleosome dense (Ou et al., 2017; Grigoryev et al., 2016), it may need to be modified before condensin can load and extrude loops. Here, we have shown that mitotic chromosome condensation and condensin loading in *Xenopu*s egg extracts require the TFIIH complex, and that TFIIH’s roles in these processes can be bypassed by reducing nucleosome density. These findings provide further support for the idea that nucleosomes inhibit condensin function, and suggest that the TFIIH complex regulates the exposure of nucleosome-free DNA where condensin can load and function (Figure 5D). Chromosome condensation depends on condensin loading as well as condensin-mediated loop extrusion, and previous experiments have suggested that these events can be separated (Goloborodko et al., 2016a; Kong et al., 2020; Kinoshita et al., 2015; Thadani et al., 2018; Ganji et al., 2018). Our results suggest that TFIIH activity promotes both steps, since TFIIH inhibition rapidly stalls the condensation process before it impacts levels of condensins on chromatin (Figure 4 and Figure 4–figure supplement 2), although it remains unclear whether TFIIH regulates these processes directly, or indirectly through removal of inhibitory proteins (see below). Since histone depletion restores both condensation and condensin loading in the absence of TFIIH activity (Figure 5A-C), it is likely that TFIIH-dependent changes to the chromatin environment are also required for condensin-mediated loop extrusion and chromatid formation. These changes could promote loop extrusion by creating additional binding sites necessary for intra-chromatid contacts and condensin processivity (Goloborodko et al., 2016a; b), or through regulation of condensin turnover (Kinoshita et al., 2015; Thadani et al., 2018). However, it is possible that TFIIH also promotes condensation in a condensin-independent manner.

During transcription initiation, the XPB subunit of TFIIH acts as a DNA translocase to help “melt” promoter DNA to allow for Pol II to engage the template strand (Nogales and Greber, 2019; Aibara et al., 2021). Our studies with triptolide, which inhibits the ATPase activity of XPB required for translocation (Titov et al., 2011; Kappenberger et al., 2020; Tomko et al., 2017), suggest that the DNA translocase function of TFIIH is important to promote condensation. TFIIH is also important for recruiting factors that promote transcription, including the histone acetyltransferase KAT2A which controls histone H3K9 acetylation (Sandoz et al., 2019), a modification that is associated with increased nucleosome turnover (Aygün et al., 2013; Zentner and Henikoff, 2013). We speculate that the TFIIH complex regulates transient cycles of DNA opening and closing at Pol II-dependent promoters that do not result in transcription but help to maintain nucleosome-free regions required for condensin loading and function (Gilchrist et al., 2010) (Figure 5D). This process may be part of or similar to mitotic transcriptional bookmarking observed in other cell types (Palozola et al., 2019). Importantly, we found that the condensation of both mouse and *Xenopus* sperm nuclei in *Xenopus* egg extracts require TFIIH activity (Figure 5–figure supplement 1A). This suggests a common fundamental mechanism that regulates condensin function. Interestingly, the TFIIH complex is present on chromosomes during mitosis in *Drosophila* embryos prior to the activation of transcription at the mid-blastula transition (Cruz-Becerra et al., 2018). Furthermore, in *Drosophila*, TFIIH interacts with the chromatin remodeling SRCAP complex (Herrera-Cruz et al., 2012), and mutation of the p52 TFIIH subunit causes chromosome condensation defects in mitotic neuroblasts (Fregoso et al., 2007). Thus, it is likely that the role of TFIIH in mitotic chromosome condensation during embryogenesis is conserved. However, the chromatin landscape during embryogenesis is quite distinct from that of somatic cells (Jukam et al., 2017; Ke et al., 2017; Du et al., 2017), and therefore factors other than or in addition to TFIIH may regulate condensin function depending on cell type. In addition, the TFIIH complex has been reported to act independently of Pol II in various non-transcriptional processes (Mizuki et al., 2007; Compe and Egly, 2016), and therefore it is possible that TFIIH promotes a condensation-competent chromatin environment on its own or in association with unknown factors independent of transcription.

Seminal work from the Hirano lab demonstrated that mitotic chromatid structure can be reconstituted *in vitro* using *Xenopus* sperm and a limited set of purified factors that include core histones, histone chaperones (nucleoplasmin, Nap1 and FACT), condensin I, and topo II (Shintomi et al., 2015; Shintomi and Hirano, 2021). The lack of TFIIH dependence in this reconstituted system could be explained by either incomplete or highly dynamic chromatinization, which would expose DNA and eliminate the need for TFIIH (Shintomi et al., 2015; Barrows and Long, 2019). Interestingly, *in vitro* chromatid reconstitution in this system specifically requires the use of an egg-specific histone (Shintomi et al., 2015; Shechter et al., 2009), histone H2A.X-F (also known as H2A.X.3), and it was suggested that this variant is a better substrate of the FACT complex chaperone than is the canonical histone H2A, which is also present in egg extracts (Shintomi et al., 2015; Wang et al., 2014). Since the FACT complex promotes both nucleosome assembly and disassembly (Zhou et al., 2020), it may act in the reconstituted system to provide the nucleosome dynamics required for condensation that TFIIH provides in egg extracts. Since the FACT histone chaperone acts in concert with Pol II during transcription (Petrenko et al., 2019), and helps promote the loading of the condensin-related cohesin complex (Garcia-Luis et al., 2019), it will be important to investigate contributions of FACT to TFIIH-dependent condensation in egg extracts and in other systems. It is also possible that TFIIH regulates the access of other non-nucleosomal factors, not present in the reconstituted system, that either impede condensin loading or serve to restrict nucleosome mobility.

Although the regulation of nucleosome occupancy may be an important factor in condensin loading and function on chromatin, it is also possible that TFIIH promotes other changes necessary for condensation. Previous work has demonstrated that changes in DNA supercoiling and/or the creation of single-stranded DNA are important for condensin loading (Sutani et al., 2015; Kim et al., 2021). Indeed, TFIIH complex can act in all three processes: it helps to maintain nucleosome-free regions (Gilchrist et al., 2010), its DNA translocase activity exposes ssDNA during transcription initiation (Aibara et al., 2021; Tomko et al., 2017; Fishburn et al., 2015), and it promotes DNA supercoiling (Aibara et al., 2021; Tomko et al., 2017). TFIIH may also be involved in the removal of other non-nucleosomal barriers to condensin function, such as histone H1 or Pol II itself (Choppakatla et al., 2021; Brandão et al., 2019; Rivosecchi et al., 2020; Pradhan et al., 2021). Understanding how TFIIH alters the chromatin environment to promote condensin function and condensation should be the focus of future efforts.

**Maintenance of the condensed chromosome state is a dynamic process regulated by TFIIH**

The mechanisms that maintain chromosome condensation are not well understood (Paulson et al., 2021). Our data show that continuous TFIIH activity is needed both to load condensin and to promote its function during the establishment and maintenance of chromosome condensation. In line with this, multiple studies have reported that the inactivation of condensin or its acute depletion from fully condensed chromosomes results in the rapid loss of condensation (Hirano and Mitchison, 1994; Piskadlo et al., 2017; Samejima et al., 2018; Nakazawa et al., 2011). Furthermore, continual ATP hydrolysis by condensin is required to maintain condensed chromosomes (Kinoshita et al., 2015; Thadani et al., 2018; Elbatsh et al., 2019). Thus, maintenance of mitotic chromosome structure is a dynamic and continuous process, and our data suggest that condensin access to DNA continues to be a critical step even after condensation is achieved. As the folding of chromatids is entropically unfavorable (Goloborodko et al., 2016b; Vasquez et al., 2016), it is worth noting that all the major factors required to promote condensation, including the XPB subunit of TFIIH, are ATPases (Paulson et al., 2021). Thus, our findings provide further support to the conclusion that mitotic chromosome assembly and maintenance are energy-dependent bi-directional processes opposed by nucleosomes and entropic forces (Shintomi and Hirano, 2021; Hirano, 2014).

**Materials and methods**

**Key Resources Table**

| **Key Resources Table** | | | | |
| --- | --- | --- | --- | --- |
| **Reagent type (species) or resource** | **Designation** | **Source or reference** | **Identifiers** | **Additional information** |
| Antibody | Anti-XPB (rabbit polyclonal) | Novus Biologicals | Cat# NB100-61060, RRID:AB_925377 | IF (1:200), WB (1:1000)  IP (0.24 µg antibody per µl beads and 0.24µg antibody per µl extract)  Immunodepletion (0.24 µg Antibody per µl beads and 1.44 µg Antibody per µl beads) |
| Antibody | Anti-XPB (mouse monoclonal) | Millipore | Cat# MABE1123 | IF (1:50) |
| Antibody | Anti-p62 (rabbit polyclonal) | Abcam | Cat# ab232982 | WB (1:400) |
| Antibody | Anti-XPF (rabbit polyclonal) | Gift of Puck Knipscheer, Hubrecht Institute  (Klein Douwel et al., 2014) | Custom | WB (1:10,000) |
| Antibody | Anti-ERCC1 (rabbit polyclonal) | Gift of Puck Knipscheer, Hubrecht Institute  (Klein Douwel et al., 2014) | Custom | WB (1:10,000)  Immunodepletion (0.24 µg antibody per 1µl of beads and 1.44µg of antibody per 1µl of extract) |
| Antibody | Anti-CAP-E Serum (rabbit polyclonal) | This study | HL7914 | Immunodepletion (25 µl of serum per µl extract and 3 µl of serum per µl of Protein A Dynabeads)  IP (3 µl of serum per µl HSS and 3 µl of serum per µl of Protein A Dynabeads)  Refer to ‘Antibody Production’ section in Methods |
| Antibody | Anti-CAP-D3 (rabbit polyclonal) | Gift of Alexei Arnaoutov and Mary Dasso, NICHD  (Bernad et al., 2011) | Custom | IF (1:100)  WB (1:400) |
| Antibody | Anti-CAP-C (rabbit polyclonal) | Gift of Alexei Arnaoutov and Mary Dasso, NICHD  (Bernad et al., 2011) | Custom | IF (1:100)  WB (1:500) |
| Antibody | Anti-CAP-E (rabbit polyclonal) | Gift of Alexei Arnaoutov and Mary Dasso, NICHD  (Bernad et al., 2011) | Custom | IF (1:100)  WB (1:1,000) |
| Antibody | Anti-CAP-G (rabbit polyclonal) | Gift of Susannah Rankin, Oklahoma Medical Research Foundation | OMRF191 | WB (1:5,000) |
| Antibody | Anti-Topo II (rabbit polyclonal) | Gift of Yoshi Azuma, University of Kansas  (Pandey et al., 2020) | Custom | IF (1:200) |
| Antibody | Anti-Histone H3 (rabbit polyclonal) | Abcam | Cat# ab1791, RRID:AB_302613 | IF (1:400)  WB (1:500) |
| Antibody | Anti-Histone (T3ph) (rabbit monoclonal) | Abcam | Cat# ab78351, RRID:AB_1566301 | WB (1:10,000) |
| Antibody | Anti-histone H4K12ac (mouse monoclonal) | Gift of Hiroshi Kimura,Tokyo Institute of Technology  (Hayashi-Takanaka et al., 2015) | Custom | Immunodepletion (0.24 µg antibody per 1 µl beads and 4.15 µg antibody per 1 µl HSS) |
| Antibody | Anti-α-Tubulin (mouse, monoclonal) | MilliporeSigma | Cat# T9026, RRID:AB_477593 | WB (1:20,000) |
| Antibody | Anti-CDK1T116Phos (rabbit polyclonal) | Cell Signaling Technology | Cat# 9114S, RRID:AB_2074652 | WB (1:500) |
| Antibody | Anti-SUPT16H (rabbit polyclonal) | Abcam | Cat# ab204343 | IF (1:100) |
| Antibody | Alexa Fluor 488 anti-rabbit IgG (donkey polyclonal) | Jackson ImmunoResearch | Cat# 711-545-152, RRID:AB_2313584 | IF (1:400) |
| Antibody | Alexa Fluor 647 anti-rabbit IgG (goat polyclonal) | Jackson ImmunoResearch | Cat# 111-605-144, RRID:AB_2338078 | IF (1:400) |
| Antibody | Alexa Fluor 488 anti-mouse IgG (goat polyclonal) | Jackson ImmunoResearch | Cat#  115-545-166, RRID:AB_2338852 | IF (1:400) |
| Antibody | IRDye 800 CW anti-rabbit IgG (H + L) (goat polyclonal) | LI-COR Biosciences | Cat# 926-32211, RRID:AB_621843 | WB (1:17,500) |
| Antibody | IRDye 680 LT anti-mouse IgG (H + L) (goat polyclonal) | LI-COR Biosciences | Cat# 926-68020, RRID:AB_10706161 | WB (1:17,500) |
| Antibody | IgG (rabbit polyclonal) | MilliporeSigma | Cat# I5006, RRID:AB_1163659 | Immunodepletion (used as control at 0.24 µg antibody per µl of beads) |
| Peptide, recombinant protein | xCAP-E C-terminal peptide | Vivitide | Custom | NH2-CSKTKERRNRMEVDK-COOH |
| Other | Protein A Dynabeads | Thermo Fisher Scientific | Cat# 10002D | Immunodepletion and Immunoprecipitation |
| Chemical compound, drug | BS^3^ (bis(sulfosuccinimidyl) suberate) | Thermo Fisher Scientific | Cat# A39266 | 4 mM |
| Chemical compound, drug | Triptolide | Tocris or MilliporeSigma | Tocris Cat# 3253  MilliporeSigma Cat# T3652 | 50 µM |
| Chemical compound, drug | α-amanitin | Tocris | Cat# 4025 | 54.4 µM |
| Chemical compound, drug | Actinomycin D | Tocris | Cat# 1229 | 0.05 µM, 0.4 µM, 4.0 µM |
| Chemical compound, drug | THZ1 | Cayman | Cat# 9002215-1 | 30 µM |
| Chemical compound, drug | DRB | Cayman | Cat# 10010302 | 100 µM |
| Chemical compound, drug | RNase A | Thermo Fisher Scientific | Cat# EN0531 | 100 µg/ml |
| Chemical compound, drug | RNase H | Thermo Fisher Scientific | Cat# EN0201 | 0.2 Units/µl |
| Other | Hoechst Stain | Thermo Fisher Scientific | Cat# H3569 | 0.5 µg/ml |
| Other | Bio-Rad Trans-Blot Nitrocellulose | Bio-Rad | Cat# 170-4159 | Western blot |
| Biological sample (*Xenopus laevis*) | *Xenopus laevis*, 9+ cm mature females, pigmented | NASCO | Cat# LM00535M | Female, adult frogs |
| Biological sample (*Xenopus laevis*) | *Xenopus laevis*, 7.5-9 cm mature males, pigmented | NASCO | Cat# LM00715MX | Male, adult frogs |
| Software, algorithm | Fiji v2.1.0/1.53f | Fiji | RRID: SCR_002285 | Image analysis and quantification |
| Software, algorithm | GraphPad Prism v9 | GraphPad | RRID: SCR_002798 | Graph creation and statistical analysis |
| Software, algorithm | NIS Elements AR 4.20.02 | Nikon Instruments | RRID: SCR_014329 | Capture and process chromatin images |
| Software, algorithm | Licor Image Studio v3.1 | LI-COR Biosciences | RRID: SCR_013715 | Capture and process Western blot images |
| Software, algorithm | Adobe Illustrator CC v25.4.3 | Adobe | RRID: SCR_010279 | Organize and prepare figures |

**Preparation of *Xenopus* egg extracts**

Metaphase-arrested *Xenopus laevis* egg extract was prepared as previously described (Haase et al., 2017). This low-speed supernatant (LSS) extract was used to prepare mitotic high-speed supernatant (HSS) extract as previously described (Maresca and Heald, 2006). Briefly, LSS extracts were supplemented with energy mix (7.5 mM creatine phosphate, 1 mM ATP, 1 mM MgCl_2_, 0.01 mM EGTA), 50 mM sucrose, and sea urchin cyclin Δ90 (Groen et al., 2011). Extracts were incubated at 21°C for 15 minutes then spun for 2 hours at 200,000 *x* *g* at 4°C. Post spin, the middle cytosolic layer was removed and spun once more for 30 minutes. The resulting supernatant was removed and aliquoted, and stored at -80°C.

**Sperm Nuclei Preparation**

*Xenopus* sperm nuclei were prepared as described previously (Maresca and Heald, 2006). Mouse (*Mus musculus*) sperm nuclei were prepared as described previously (Shintomi et al., 2017), with the following modifications. Sperm were isolated from the cauda epididymis of ~ 6-month old C57BL/6J mice. To detach sperm tails from the nuclei-containing sperm heads, isolated sperm were resuspended in 2 ml of tissue-culture grade trypsin/EDTA (Thermo) and incubated for 5 minutes in a 37°C water bath. Digestion was halted by resuspension of the mixture in 1X PBS containing 10X LPC (10 mg/mL each of leupeptin, pepstatin, and chymostatin). To isolate sperm heads from tails, the digestion mixture (resuspended in 1X MH buffer [20 mM HEPES-KOH, pH 7.7, 2 mM MgCl_2_] + 1.8M sucrose) was layered onto a sucrose step gradient (containing 2.05 and 2.2 M sucrose, in 1X MH buffer) and centrifuged for 45 minutes at 93,000 *x g* at 2°C. After washing the interfaces with 1X MH buffer, the pellet containing sperm heads was resuspended in 1X MH + 250 mM sucrose. Sperm nuclei were demembranated as described previously (Shintomi et al., 2017), and protamines were reduced in a solution of 1X MH, 250 mM sucrose, 0.4% BSA, 1X LPC, and 50 mM DTT for 2 hours in a room temperature water bath. Sperm were washed, resuspended, and stored as described.

**Chromatid assembly reactions and drug treatments**

To monitor the assembly of chromatids, HSS egg extracts were first diluted with an equal volume of XBE5 buffer (10 mM HEPES-KOH [pH 7.7], 100 mM KCl, 5 mM EGTA, 5 mM MgCl_2_, 50 mM sucrose) and supplemented with energy mix. Sperm nuclei isolated from *Xenopus* or mouse were then added to the diluted HSS extracts at a final concentration of 2,000 sperm/μl, and incubated for the indicated times at 21°C. Samples were taken at indicated timepoints for analysis via microscopy and Western blot.

For inhibition experiments, chromatin assembly reactions were allowed to proceed for 25 minutes to allow for protamine exchange with maternal histones, and then drugs or an equal volume of DMSO were added and the extracts were further incubated at 21°C for the indicated times. The final concentration of inhibitors was: 54.4 μM α-amanitin (Tocris), 50 μM triptolide (TPL; Tocris or Sigma), 30 μM THZ1 (Cayman), and 100 μM DRB (Cayman).

**RNase treatments**

To determine the role of RNA in chromosome condensation, HSS extracts were either mock-treated (RNase-free water) or treated with RNase A (Thermo Fisher Scientific) at a final concentration of 100 μg/ml and incubated at 21°C for 30 minutes prior to the addition of sperm. Sperm nuclei were then added to a final concentration of 2,000 sperm/μl and the reaction was further incubated at 21°C for up to 180 minutes in the presence of active RNase A. Samples were taken at indicated timepoints and processed for microscopy analysis. To analyze total RNA levels, RNA was isolated from 100 μl of HSS extracts according to the RNeasy kit “RNA clean up” protocol (Qiagen), precipitated in the presence of glycogen (MilliporeSigma), and resuspended in 2 μl of RNase-free water. Samples were then diluted with RNA loading buffer (95% formamide, 10 mM EDTA pH 8, 0.025% SDS, bromophenol blue) and run on a 1% agarose gel in 0.5X TBE, and visualized with SYBR safe DNA gel stain (Thermo Fisher Scientific). Note that total RNA levels in HSS extracts are low compared to LSS extracts due to the lower concentration of ribosomal RNAs (Groen et al., 2011).

To degrade RNA:DNA hybrids, HSS extracts were treated with RNase H (Thermo Fisher Scientific) or heat-inactivated RNase H (65°C for 20 minutes) at a final concentration of 0.2 U/μl. Sperm nuclei were then added to a final concentration of 2,000 sperm/μl and the reaction was incubated at 21°C for up to 180 minutes. Samples were taken at indicated timepoints and processed for microscopy analysis.

**Antibody Production**

CAP-E antibodies were raised against a C-terminal peptide of *Xenopus* laevis CAP-E (CSKTKERRNRMEVDK; synthesized by Vivitde) (Hirano et al., 1997). The peptide was then coupled to keyhole limpet hemocyanin protein and used to immunize rabbits (Labcorp).

**Immunodepletion of HSS extracts**

Control rabbit IgG (MilliporeSigma, I5006), anti-XPB (Novus Biologicals, NB100-61060), anti-ERCC1 (a kind gift of Puck Knipscheer, Hubrecht Institute, (Klein Douwel et al., 2017; 2014)), or anti-histone H4K12ac (a kind gift of Hiroshi Kimura, Tokyo Institute of Technology, (Zierhut et al., 2014)) were bound and crosslinked to Protein-A Dynabeads as previously described (Haase et al., 2017). HSS extract was incubated with antibody-coupled beads at 4°C for 30 minutes with occasional mixing, followed by two additional rounds of depletion. For CAP-E depletion, CAP-E serum or pre-immune serum (“Mock”) were bound to Protein-A Dynabeads without crosslinking, and HSS extract was incubated with antibody-coupled beads at 4ºC for 45 minutes, followed by one additional round of depletion. The volume ratio of beads to extract for each depletion were as follows: CAP-E serum (4:1), anti-XPB (2:1), anti-ERCC1 (2:1), and anti-H4K12ac (5.76:1). After depletion, the extract was used for chromatid assembly reactions as indicated.

**Immunoprecipitations**

To provide samples for the analysis of XPB interaction partners by mass spec, XPB antibody (Novus Biologicals, NB100-61060) was incubated with Protein-A Dynabeads (Thermo Fisher Scientific, 10001D) and then crosslinked with BS^3^ (Thermo Fisher Scientific, A39266) at a ratio of 0.24 μg of antibody per μl of beads. 300 μl of HSS extract was then incubated with an equal volume of anti-XPB beads for 60 minutes at 4°C, with occasional mixing. Anti-XPB beads were then captured and washed four times with wash buffer (1X PBS, 0.01% Tween-20), and boiled in 2X sample buffer for 5 min at 95°C.

For XPB and CAP-E immunoprecipitations, XPB antibody (Novus Biologicals, NB100-61060), control IgG antibody (MilliporeSigma, I5006), anti-CAP-E serum, or pre-immune (“Mock”) serum were incubated with Protein-A Dynabeads at a ratio of 0.24 μg XPB or IgG antibody per μl of beads or 3 μl anti-CAP-E or pre-immune serum per μl of beads, and subsequently crosslinked with BS^3^. 75 μl of HSS extract was then incubated with an equal volume of antibody-bound beads for 60 minutes at 4°C, with occasional mixing. Antibody-bound beads were then captured and washed four times with wash buffer (1X PBS, 0.01% Tween-20), and boiled in 2X sample buffer for 10 min at 70°C.

**Mass Spectrometry**

The immunoprecipitated proteins were separated by gel electrophoresis. Each lane was divided into twelve fractions and the proteins were in-gel digested with trypsin (Thermo Fisher Scientific) at 37 °C for 16 h, as described (Shevchenko et al., 2006). Dried peptides were separated on a 75 µm x 15 cm, 2 µm Acclaim PepMap reverse phase column (Thermo) at 300 nL/min using an UltiMate 3000 RSLCnano HPLC (Thermo Fisher Scientific). Peptides were eluted into a Thermo Orbitrap Fusion mass spectrometer using a linear gradient from 96% mobile phase A (0.1% formic acid in water) to 55% mobile phase B (0.1% formic acid in acetonitrile) over 30 minutes. Parent full-scan mass spectra were collected in the Orbitrap mass analyzer set to acquire data at 120,000 FWHM resolution; ions were then isolated in the quadrupole mass filter, fragmented within the HCD cell (HCD normalized energy 32%, stepped ± 3%), and the product ions were analyzed in the ion trap. Proteome Discoverer 2.4 (Thermo Fisher Scientific) was used to search the data against *Xenopus laevis* proteins from the PHROG database (Wühr et al., 2014; Presler et al., 2017) using SequestHT. The search was limited to tryptic peptides, with maximally two missed cleavages allowed. Cysteine carbamidomethylation was set as a fixed modification, with methionine oxidation and serine/threonine phosphorylation set as variable modifications. The precursor mass tolerance was 10 ppm, and the fragment mass tolerance was 0.6 Da. The Percolator node was used to score and rank peptide matches using a 1% false discovery rate.

**Western blots**

Primary antibodies were diluted in Licor blocking solution with a final Tween-20 concentration of 0.1%. The following antibodies and antibody dilutions were used: anti-CDK1T161ph (9114S, 1:500, Cell Signaling Technology), anti-XPB (NB100-61060, 1:1000; Novus Biologicals), anti-p62 (ab232982, 1:400; Abcam), anti-α-Tubulin (DM1, 1:20,000; MiiliporeSigma), anti-H3 (ab1791, 1:500; Abcam), anti-histone H3T3ph (ab78351, 1:10,000, Abcam), anti-XPF (a kind gift of Puck Knipscheer, Hubrecht Institute, (Klein Douwel et al., 2017; 2014); 1:10,000), anti-ERCC1 (a kind gift of Puck Knipscheer, Hubrecht Institute, (Klein Douwel et al., 2017; 2014); 1:10,000), anti-CAP-E (a kind gift of Alexei Arnaoutov and Mary Dasso, NICHD (Bernad et al., 2011); 1:1000), anti-CAP-C (a kind gift of Alexei Arnaoutov and Mary Dasso, NICHD (Bernad et al., 2011; 1:500), anti-CAP-D3 (a kind gift of Alexei Arnaoutov and Mary Dasso, NICHD (Bernad et al., 2011); 1:400), and anti-CAP-G (a kind gift of Susannah Rankin, OMRF; 1:5000). Secondary antibodies (Licor) were used at a 1:17,500 dilution (goat anti-rabbit 800 nm and goat anti-mouse 680 nm), and membranes were scanned on the Odyssey imaging system (Licor).

**Microscopy and Fluorescence Quantification**

To immunostain chromatin structures, extracts samples were fixed by diluting ten-fold with KMH buffer (20 mM Hepes-KOH pH 7.7, 100 mM KCl, 2.5mM MgCl2) containing 4% formaldehyde and 0.1% Triton X-100, and incubated for 15 minutes at room temperature. To isolate chromatids/nuclei, the fixed reactions were layered onto cushions of KMH + 30% glycerol in a 24-well plate, which contained poly-L-lysine–coated coverslips (No. 1) at the bottom of each well, and then spun at 4,614 x *g* for 12.5 min at 18°C in a centrifuge (Eppendorf 5810R). Cushion was removed and the coverslips were washed with TBS-TX twice for 5 minutes each, then blocked with Abdil (TBS + 0.1% Tween20 + 2% BSA + 0.1% sodium azide) for 30 minutes at 4°C, then incubated in primary at 4°C overnight unless otherwise noted. Chromatids/nuclei were then washed three times with AbDil and then incubated with secondary antibody for 2 hours. All washes and antibody dilutions were carried out with AbDil buffer. Chromatids/nuclei were stained with Hoechst 33258 prior to mounting in 80% glycerol + PBS medium. The following antibodies were used at the indicated dilutions: XPB (Millipore, MABE1123) 1:50, XPB (Novus Biologicals NB100-61060) 1:200, CAP-D3 (a kind gift of Alexei Arnaoutov and Mary Dasso, NICHD; (Bernad et al., 2011)) 1:100, CAP-C (Bernad et al., 2011) 1:100, CAP-E (Bernad et al., 2011) 1:100, Topo II (a kind gift of Yoshi Azuma, University of Kansas; (Pandey et al., 2020)) 1:200, SUPT16H (Abcam, ab204343) 1:100, histone H3 (Abcam, ab1791) 1:400. Alexa 488 (anti-rabbit; Jackson Immunoresearch), Alexa 488 (anti-mouse; Jackson Immunoresearch), and Alexa 647 (anti-rabbit; Jackson Immunoresearch) conjugated secondary antibodies were used at a 1:400 dilution for immunofluorescence detection.

All immunofluorescence was imaged with 0.2 μm step size using an Eclipse Ti (Nikon) comprised of a Nikon Plan Apo x100/1.45, oil immersion objective, a PlanApo x40/0.95 objective, and a Hamamatsu Orca-Flash 4.0 camera. Images were captured and processed using NIS Elements AR 4.20.02 software (Nikon), and analyzed in Fiji ImageJ. The acquired Z-sections of 0.2 μm each were converted to a maximum projection using NIS Elements and Fiji. Intensity of chromatin bound proteins was measured using Fiji by centering 9 x 9 and 13x13 pixel regions along multiple points of individualized chromatids and averaging the maximum intensity of each sample point to determine average intensity per chromatid. In cases where condensation was inhibited, chromatin masses were sampled to determine an average intensity. To correct for background fluorescence, the difference in intensities between the two regions was determined, and then made proportionate to the smaller region. This background value was then subtracted from the smaller region to determine chromatin bound protein intensity with background correction as previously reported (Hoffman et al., 2001).

**Condensation Parameter**

To quantify condensation of chromatin, 20 chromatin structures per condition were sampled with a 38 by 50 pixel region. Pixel intensities were recorded and binned. To measure the degree of condensation, the percentage of pixels below a threshold of 35% of the maximum image intensity were determined (Maddox et al., 2006). This percentage serves as the condensation parameter, and represents the changes in distribution of fluorescence intensity that result from condensation.

**Statistical Analysis**

All analyses were performed with a minimum number of 50 chromatids, or chromatin masses in cases where condensation was not achieved. This sample size was chosen to ensure a high (>90%) theoretical statistical power in order to generate reliable P values. All graphs and statistical analysis were prepared with GraphPad Prism. Fluorescence values from experimental conditions were compared to control conditions using an ordinary one-way ANOVA with Tukey’s multiple comparison tests to determine significance. All graphs show the mean with error bars representing the S.D. unless otherwise indicated. At least two biological replicates (using two different preparations of egg extracts) were performed for each experiment and each confirmed similar behavior amongst the replicates.

**Acknowledgements**

We thank Takashi Akera, Chongyi Chen, and Michael Lichten for critical reading of the manuscript, and members of the LBMB for comments and support, and A. Arnaoutov, Y. Azuma, M. Dasso, H. Kimura, P. Knipscheer, and A. Nussenzweig for kindly providing reagents. We thank Jacob Paiano for assistance with mouse sperm purification, and Mia Rosenfeld for initial work with inhibitors.

**Figure 1. The TFIIH complex is required for chromosome condensation in Xenopus egg extracts.** (**A**) Top: Schematic of chromatid assembly in M-phase high-speed supernatant (HSS) extract. Bottom: Representative fluorescence images of fixed samples from a chromatid assembly reaction taken at indicated timepoints after sperm addition. Chromatin was stained with Hoechst. (**B**) Representative fluorescence images of chromatid assembly at steady state (180 mins after sperm addition) in the presence of indicated inhibitors. Each drug or DMSO control was added at 25 mins to exclude effects on the protamine-histone exchange process. Final inhibitor concentrations: Triptolide (50 μM), DRB (100 μM), and a-amanitin (54 μM). See Figure 1**–**figure supplement 1A for CAP-E depletion. Mean condensation parameters for each condition are indicated in lower left corner of each image. (**C**) Scatter plot of the percentage of pixels below a threshold of 35% of image maximum fluorescence intensity (the condensation parameter), which measures the progressive change in the fluorescence intensity distribution that occurs during condensation, for each condition. Error bars represent SD, and the mean values are indicated. n = 20 structures for each condition. Two biological replicates were performed, quantified structures are from a single experiment. (**D**) Schematic of the TFIIH complex. TFIIH core complex is green, CAK subcomplex in red. (**E**) Western blot for XPB, p62, and Tubulin in IgG or XPB depleted extracts. (**F**) TFIIH complex members that interact with XPB in M-phase HSS extract, as identified by mass spectrometry. Purifications were performed in the absence of chromatin. PSMs = peptide spectrum matches. (**G**) Representative fluorescence images of chromatid assembly 90 mins after sperm addition in IgG or XPB depleted extracts. Chromatin was stained with Hoechst. (**H**) Western blot for XPF, ERCC1, and Tubulin in IgG or ERCC1 depleted extracts. (**I**) Representative fluorescence images of chromatid assembly 180 mins after sperm addition in IgG or ERCC1 depleted extracts. Chromatin was stained with Hoechst.

**Figure 1–source data 1.** Source data for Figure 1E and H.

**Figure 1–figure supplement 1. Immunodepletions and the effects of RNases and various transcription inhibitors on chromosome condensation and cell cycle state.** (**A**) Western blot for CAP-E, XPB, and Tubulin in Mock or CAP-E depleted extracts. Dilution series of Mock-depleted extracts was used to calculate the % depletion of CAP-E using quantitative fluorescence. (**B**) 1% agarose gel stained with SYBR safe stain showing RNase A dependent removal of total RNAs from high-speed supernatant egg extracts. (**C**) Representative fluorescence images of chromatid assembly at steady state (180 mins after sperm addition) in the presence of indicated buffers and enzymes. See methods for details. Note that the high salt in the RNase H buffer causes morphological changes to chromatids regardless of activity. Mean condensation parameters for each condition are indicated in lower left corner of each image. (**D**) Western blot for XPB and Tubulin in IgG or XPB depleted extracts. Dilution series of IgG-depleted extracts was used to calculate the % depletion of XPB using quantitative fluorescence. (**E**) Western blot for XPF, ERCC1, and Tubulin in IgG or ERCC1 depleted extracts. Dilution series of IgG-depleted extracts was used to calculate the % depletion of ERCC1 and XPF using quantitative fluorescence. (**F**) Representative fluorescence images of chromatid assembly at steady state (180 mins after sperm addition) in the presence of DMSO, 30 μM CDK7 inhibitor THZ1, or 30 μM THZ1 and 50 μM triptolide (TPL). (**G**) Triptolide does not affect CDK1 activation or maintenance of M phase. Western blot for CDK1 T161ph, histone H3T3ph, and Tubulin in M phase HSS extracts in the presence of DMSO or 50 μM triptolide. Both CDK1T161ph (a CDK7 substrate) and H3T3ph are only highly phosphorylated in M phase, and their appearance is not affected by triptolide treatment.

**Figure 1–figure supplement 1–source data 1.** Source data for Figure 1–figure supplement 1A, B, D, E, and G.

**Figure 1–figure supplement 2. Effects of various transcription inhibitors on RNA Polymerase II C-terminal phosphorylation at centromeres.** (**A**) Representative immunofluorescence images of *Xenopus* sperm nuclei incubated in HSS extracts for 180 min. DMSO was added at 25 min as a control. Chromatids were labeled with Hoechst, either anti-Pol II S5ph (paused) or anti-Pol II S2ph (elongating), and co-stained with anti-CENP-A antibodies. (**B**) Representative immunofluorescence images of *Xenopus* sperm nuclei incubated in DMSO, triptolide (TPL), THZ1, DRB, or alpha-amanitin treated HSS extracts for 180 min. All inhibitors were added 25 min after sperm addition. Chromatids were labeled with Hoechst, anti-Pol II S5ph, or anti-Pol II S2ph antibodies.

**Figure 1–figure supplement 3. Condensation parameters.** (**A-D**) Scatter plots of the percentage of pixels below a threshold of 35% of image maximum fluorescence intensity (the condensation parameter), for indicated conditions in Figure 1 and Figure 1**–**figure supplement 1. Error bars represent SD, and the mean values are indicated. n = 20 structures for each condition. Two biological replicates were performed, quantified structures are from a single experiment.

**Figure 2**. **Continuous TFIIH activity is required to maintain chromosome structure**. (**A**) Representative fluorescence images of chromatid assembly reactions sampled at indicated timepoints. DMSO or triptolide (TPL) were added at indicated times, immediately after sample retrieval for imaging. Chromatin was stained with Hoechst. Red boxes indicate representative images of decondensed chromosomes after triptolide addition. Mean condensation parameters for each condition are indicated in lower left corner of each image, see Figure 2–figure supplement 1A for more details. (**B**) Representative fluorescence images of chromatid masses fixed and spun onto coverslips 215 mins after sperm addition, subjected to the indicated manipulations. Nuclei were incubated in extracts for 180 mins to allow for the formation of fully condensed chromatids, followed by addition of DMSO or triptolide. After 30 mins of incubation with DMSO/triptolide, reactions were diluted 10-fold with extract containing DMSO or triptolide, and further incubated for 5 min before taking samples for imaging. See Figure 2–figure supplement 2 for additional controls. Chromatin was stained with Hoechst. (**C**) Scatter plots of the percentage of pixels below a threshold of 35% of image maximum fluorescence intensity (the condensation parameter), for each condition in (B). Error bars represent SD, and the mean values are indicated. n = 20 structures for each condition. Two biological replicates were performed, quantified structures are from a single experiment. (**D**) Schematic illustrating the effects of triptolide addition and dilution on chromosome structure maintenance and reformation.

**Figure 2–figure supplement 1. Condensation parameters of condensation kinetics after DMSO or Triptolide treatment.** (**A**) Scatter plots of the percentage of pixels below a threshold of 35% of image maximum fluorescence intensity (the condensation parameter), for each drug condition and indicated time point. Error bars represent SD, and the mean values are indicated. n = 20 structures for each condition. Two biological replicates were performed, quantified structures are from a single experiment.

**Figure 2–figure supplement 2. Continuous TFIIH activity is required to maintain chromosome structure and condensin levels on chromatin.** (**A**) Representative fluorescence images of *Xenopus* sperm nuclei incubated in extracts at indicated timepoints. DMSO or Triptolide were added 25 mins after addition of nuclei, and extracts were diluted 10-fold with extract containing DMSO or Triptolide at 210 mins after nuclei addition, and further incubated for 5 min before imaging. Chromatin was stained with Hoechst. (**B**) Scatter plots of the percentage of pixels below a threshold of 35% of image maximum fluorescence intensity (the condensation parameter), for each condition in (A). Error bars represent SD, and the mean values are indicated. n = 20 structures for each condition. Two biological replicates were performed, quantified structures are from a single experiment. (**C**) Condensin levels decrease after treatment of condensed chromatids with triptolide for 35 mins. Left: Schematic of experimental conditions, top condition is from (A), bottom condition is from Figure 2B. Middle: Representative immunofluorescence images of *Xenopus* sperm nuclei from indicated experiments. Imaging was performed 215 mins after nuclei addition. Chromatids were labelled with Hoechst and anti-CAP-C (condensin I & II) antibodies. Right: Quantification of fluorescence intensity of CAP-C on chromatin, normalized to DMSO. n = 50 structures for each condition. Error bars represent SD, and asterisks indicate a statistically significant difference (*, P < 0.001). A.U., arbitrary units. Two biological replicates were performed, quantified structures are from a single experiment.

**Figure 3. The TFIIH complex is required for the enrichment of condensins on chromosomes.** (**A**) Representative immunofluorescence images of *Xenopus* sperm nuclei incubated with DMSO or triptolide (TPL) treated extracts for 180 min. Chromatids were labelled with Hoechst and anti-XPB, anti-CAP-D3 (condensin II), anti-CAP-C (condensin I & II), anti-CAP-E (condensin I & II), anti-SUPT16H (FACT complex), or anti-Topo II antibodies. Images of individual chromatids are shown in insets. (**B**) Quantification of fluorescence intensity of indicated proteins from experiment in (A), normalized to DMSO. n = 50 structures for each condition. Error bars represent SD, and asterisks indicate a statistically significant difference (*, P < 0.001), NS indicates no statistical significance. A.U., arbitrary units. Two biological replicates were performed, quantified structures are from a single experiment.

**Figure 3–figure supplement 1. The TFIIH complex is required for the enrichment of condensins on chromosomes.** (**A**) Left: Representative immunofluorescence images of *Xenopus* sperm nuclei incubated with Mock or CAP-E depleted extracts for 180 min. Chromatids were labelled with Hoechst and anti-XPB antibodies. Right: Quantification of fluorescence intensity of XPB on chromatin, normalized to Mock depleted condition. n = 24 structures for each condition. Error bars represent SD, and “NS” indicates no statistically significant difference (P > 0.001). A.U., arbitrary units. Two biological replicates were performed, quantified structures are from a single experiment. (**B**) Western blot for indicated proteins that copurify with anti-XPB or IgG beads isolated from HSS extracts. IP, immunoprecipitate. (**C**) Western blot for indicated proteins that copurify with anti-CAP-E or Mock-treated beads isolated from HSS extracts. For condensin subunit detection, the blot was probed simultaneously with the indicated antibodies. Beads were coupled to either anti-CAP-E or pre-immune serum (“Mock”) and processed as indicated in the methods. IP, immunoprecipitate. (**D**) Representative immunofluorescence images of *Xenopus* sperm nuclei incubated with HSS extracts for 180 min. Chromatids were labelled with Hoechst and co-stained with anti-XPB and anti-CAP-E antibodies. Images of three representative individual chromatids are shown.

**Figure 3–figure supplement 1–source data 1.**Source data for Figure 3–figure supplement 1B and C.

**Figure 4. Triptolide perturbs condensation prior to its effects on condensin levels.** (**A**) Schematic of assay to test the timing of triptolide-induced defects in condensation and condensin levels. After a twenty-five minute incubation, either DMSO or triptolide (TPL) was added, and samples were taken at indicated times and processed for immunofluorescence. (**B**) Representative immunofluorescence images of DMSO or triptolide treated extracts at indicated timepoints. Chromatids were labelled with Hoechst and anti-CAP-E (condensin I & II). Note that condensation is already lost in in the first timepoint after triptolide addition, as in Figure 2A. Mean condensation parameters for each condition are indicated in lower left corner of each image. (**C**) Quantification of fluorescence intensity of CAP-E from experiment in (B), normalized to the 180 min DMSO-treated sample. n = 50 structures for each condition. Error bars represent SD, and asterisks indicate a statistically significant difference (*, P < 0.001). A.U., arbitrary units. Two biological replicates were performed, quantified structures are from a single experiment.

**Figure 4–figure supplement 1. Condensation parameters of condensation kinetics after DMSO or Triptolide treatment.** (**A**) Scatter plots of the percentage of pixels below a threshold of 35% of image maximum fluorescence intensity (the condensation parameter), for each drug condition and indicated time point. Error bars represent SD, and the mean values are indicated. n = 20 structures for each condition. Two biological replicates were performed, quantified structures are from a single experiment.

**Figure 4–figure supplement 2. Triptolide perturbs condensation prior to its effects on condensin levels.** (**A**) Left: Representative immunofluorescence images of DMSO or triptolide treated extracts at indicated timepoints. Chromatids were labelled with DAPI and anti-CAP-E (condensin I & II). Right: Quantification of fluorescence intensity of CAP-E, normalized to the 180 min DMSO-treated sample. This data is also shown in Figure 4B in grayscale. (**B**) Left: Representative immunofluorescence images of DMSO or triptolide treated extracts at indicated timepoints. Chromatids were labelled with DAPI and anti-CAP-D3 (condensin II). Right: Quantification of fluorescence intensity of CAP-D3, normalized to the 180 min DMSO-treated sample. (**C**) Left: Representative immunofluorescence images of DMSO or triptolide treated extracts at indicated timepoints. Chromatids were labelled with DAPI and anti-XPB (TFIIH complex). Right: Quantification of fluorescence intensity of XPB, normalized to the 180 min DMSO-treated sample. n = 50 structures for each condition. Error bars represent SD, and asterisks indicate a statistically significant difference (*, P < 0.001). A.U., arbitrary units. Two biological replicates were performed, quantified structures are from a single experiment.

**Figure 5. Partial histone H3/H4 depletion rescues chromosome condensation in the absence of TFIIH activity.** (**A**) Representative fluorescence images of mouse sperm nuclei incubated with ∆IgG or ∆H4K12ac extracts and spun onto coverslips at indicated timepoints. DMSO or Triptolide were added at t=25’, immediately after sample acquisition for imaging. Chromatin was stained with Hoechst. Mean condensation parameters for each condition are indicated in lower left corner of each image. (**B**) Representative immunofluorescence images of mouse sperm nuclei incubated with ∆IgG or ∆H4K12ac extracts for 180 min. DMSO or triptolide were added at t=25’. Chromatids were labelled with anti-XPB, anti-CAP-E (condensin I & II), or anti-histone H3, and co-stained with Hoechst. (**C**) Quantification of fluorescent intensity of indicated proteins from (B), normalized to DMSO. n = 50 structures for each condition. Error bars represent SD, and asterisks indicate a statistically significant difference (*, P < 0.001). A.U., arbitrary units. Two biological replicates were performed, quantified structures are from a single experiment. (**D**) Model depicting the role of the TFIIH complex in regulating condensin dynamics. Our data are consistent with a model in which TFIIH alters the chromatin environment to allow condensins to load and extrude loops of DNA.

**Figure 5–figure supplement 1. Effects of triptolide and histone depletion on condensation using mouse sperm.** (**A**) Representative fluorescence images of chromatid assembly at steady state with mouse sperm nuclei in egg extracts (180 mins after sperm nuclei addition) in the presence of indicated inhibitors. Triptolide or DMSO control was added at 25 mins after nuclei addition. Triptolide was added at 50 μM. (**B**) Western blot for histone H3, CAP-E, and XPB in histone H4K12ac or IgG depleted extracts in the presence of triptolide (TPL) or DMSO. Relative percentage of protein remaining after indicated treatments is shown.

**Figure 5–figure supplement 1–source data 1.** Source data for Figure 5–figure supplement 1B.

**Figure 5–figure supplement 2. Condensation parameters of condensation kinetics after DMSO or Triptolide treatment.** (**A**) Scatter plots of the percentage of pixels below a threshold of 35% of image maximum fluorescence intensity (the condensation parameter), for each drug condition and indicated time point, see Figure 5A for images. Error bars represent SD, and the mean values are indicated. n = 20 structures for each condition. Two biological replicates were performed, quantified structures are from a single experiment.

**References**

Aibara, S., S. Schilbach, and P. Cramer. 2021. Structures of mammalian RNA polymerase II pre-initiation complexes. *Nature*. 594:124–128. doi:10.1038/s41586-021-03554-8.

Akhtar, M.S., M. Heidemann, J.R. Tietjen, D.W. Zhang, R.D. Chapman, D. Eick, and A.Z. Ansari. 2009. TFIIH kinase places bivalent marks on the carboxy-terminal domain of RNA polymerase II. *Mol. Cell*. 34:387–393. doi:10.1016/j.molcel.2009.04.016.

Amodeo, A.A., D. Jukam, A.F. Straight, and J.M. Skotheim. 2015. Histone titration against the genome sets the DNA-to-cytoplasm threshold for the Xenopus midblastula transition. *Proc. Natl. Acad. Sci. U.S.A.* 112:E1086–95. doi:10.1073/pnas.1413990112.

Aygün, O., S. Mehta, and S.I.S. Grewal. 2013. HDAC mediated suppression of histone turnover promotes epigenetic stability of heterochromatin. *Nat Struct Mol Biol*. 20:547–554. doi:10.1038/nsmb.2565.

Bakhrebah, M., T. Zhang, J.R. Mann, P. Kalitsis, and D.F. Hudson. 2015. Disruption of a conserved CAP-D3 threonine alters condensin loading on mitotic chromosomes leading to chromosome hypercondensation. *J. Biol. Chem.* 290:6156–6167. doi:10.1074/jbc.M114.627109.

Ball, A.R., J.A. Schmiesing, C. Zhou, H.C. Gregson, Y. Okada, T. Doi, and K. Yokomori. 2002. Identification of a chromosome-targeting domain in the human condensin subunit CNAP1/hCAP-D2/Eg7. *Mol. Cell. Biol.* 22:5769–5781. doi:10.1128/MCB.22.16.5769-5781.2002.

Barrows, J.K., and D.T. Long. 2019. Cell-free transcription in Xenopus egg extract. *J. Biol. Chem.* 294:19645–19654. doi:10.1074/jbc.RA119.011350.

Bernad, R., P. Sánchez, T. Rivera, M. Rodríguez-Corsino, E. Boyarchuk, I. Vassias, D. Ray-Gallet, A. Arnaoutov, M. Dasso, G. Almouzni, and A. Losada. 2011. Xenopus HJURP and condensin II are required for CENP-A assembly. 192:569–582. doi:10.1083/jcb.201005136.

Bernard, P., and V. Vanoosthuyse. 2015. Does transcription play a role in creating a condensin binding site? *Transcription*. 6:12–16. doi:10.1080/21541264.2015.1012980.

Blower, M.D. 2016. Centromeric Transcription Regulates Aurora-B Localization and Activation. *Cell Rep*. 15:1624–1633. doi:10.1016/j.celrep.2016.04.054.

Brandão, H.B., P. Paul, A.A. van den Berg, D.Z. Rudner, X. Wang, and L.A. Mirny. 2019. RNA polymerases as moving barriers to condensin loop extrusion. *Proc. Natl. Acad. Sci. U.S.A.* 116:20489–20499. doi:10.1073/pnas.1907009116.

Brykczynska, U., M. Hisano, S. Erkek, L. Ramos, E.J. Oakeley, T.C. Roloff, C. Beisel, D. Schübeler, M.B. Stadler, and A.H.F.M. Peters. 2010. Repressive and active histone methylation mark distinct promoters in human and mouse spermatozoa. *Nat. Struct. Mol. Biol.* 17:679–687. doi:10.1038/nsmb.1821.

Chen, H., L.C. Einstein, S.C. Little, and M.C. Good. 2019. Spatiotemporal Patterning of Zygotic Genome Activation in a Model Vertebrate Embryo. *Dev. Cell*. 49:852–866.e7. doi:10.1016/j.devcel.2019.05.036.

Choppakatla, P., B. Dekker, E.E. Cutts, A. Vannini, J. Dekker, and H. Funabiki. 2021. Linker histone H1.8 inhibits chromatin-binding of condensins and DNA topoisomerase II to tune chromosome length and individualization. *Elife*. 10. doi:10.7554/eLife.68918.

Chu, L., Z. Liang, M. Mukhina, J. Fisher, N. Vincenten, Z. Zhang, J. Hutchinson, D. Zickler, and N. Kleckner. 2020. The 3D Topography of Mitotic Chromosomes. *Mol. Cell*. 79:902-916.e6. doi:10.1016/j.molcel.2020.07.002.

Compe, E., and J.-M. Egly. 2012. TFIIH: when transcription met DNA repair. *Nat. Rev. Mol. Cell Biol.* 13:343–354. doi:10.1038/nrm3350.

Compe, E., and J.-M. Egly. 2016. Nucleotide Excision Repair and Transcriptional Regulation: TFIIH and Beyond. *Annual Review of Biochemistry*. 85:265–290. doi:10.1146/annurev-biochem-060815-014857.

Cruz-Becerra, G., S. Valerio-Cabrera, M. Juárez, A. Bucio-Mendez, and M. Zurita. 2018. TFIIH localization is highly dynamic during zygotic genome activation in Drosophila, and its depletion causes catastrophic mitosis. *J. Cell. Sci.* 131:jcs211631. doi:10.1242/jcs.211631.

D'Ambrosio, C., C.K. Schmidt, Y. Katou, G. Kelly, T. Itoh, K. Shirahige, and F. Uhlmann. 2008. Identification of cis-acting sites for condensin loading onto budding yeast chromosomes. *Genes Dev.* 22:2215–2227. doi:10.1101/gad.1675708.

Davidson, I.F., and J.-M. Peters. 2021. Genome folding through loop extrusion by SMC complexes. *Nat. Rev. Mol. Cell Biol.* 22:1–20. doi:10.1038/s41580-021-00349-7.

Dowen, J.M., S. Bilodeau, D.A. Orlando, M.R. Hübner, B.J. Abraham, D.L. Spector, and R.A. Young. 2013. Multiple structural maintenance of chromosome complexes at transcriptional regulatory elements. *Stem Cell Reports*. 1:371–378. doi:10.1016/j.stemcr.2013.09.002.

Du, Z., H. Zheng, B. Huang, R. Ma, J. Wu, X. Zhang, J. He, Y. Xiang, Q. Wang, Y. Li, J. Ma, X. Zhang, K. Zhang, Y. Wang, M.Q. Zhang, J. Gao, J.R. Dixon, X. Wang, J. Zeng, and W. Xie. 2017. Allelic reprogramming of 3D chromatin architecture during early mammalian development. *Nature*. 547:232–235. doi:10.1038/nature23263.

Earnshaw, W.C., and M.M. Heck. 1985. Localization of topoisomerase II in mitotic chromosomes. *J. Cell Biol.* 100:1716–1725. doi:10.1083/jcb.100.5.1716.

Earnshaw, W.C., and U.K. Laemmli. 1983. Architecture of metaphase chromosomes and chromosome scaffolds. *J. Cell Biol.* 96:84–93. doi:10.1083/jcb.96.1.84.

Ebmeier, C.C., B. Erickson, B.L. Allen, M.A. Allen, H. Kim, N. Fong, J.R. Jacobsen, K. Liang, A. Shilatifard, R.D. Dowell, W.M. Old, D.L. Bentley, and D.J. Taatjes. 2017. Human TFIIH Kinase CDK7 Regulates Transcription-Associated Chromatin Modifications. *Cell Rep*. 20:1173–1186. doi:10.1016/j.celrep.2017.07.021.

Elbatsh, A.M.O., E. Kim, J.M. Eeftens, J.A. Raaijmakers, R.H. van der Weide, A. García-Nieto, S. Bravo, M. Ganji, J. Uit de Bos, H. Teunissen, R.H. Medema, E. de Wit, C.H. Haering, C. Dekker, and B.D. Rowland. 2019. Distinct Roles for Condensin's Two ATPase Sites in Chromosome Condensation. *Mol. Cell*. 76:724–737.e5. doi:10.1016/j.molcel.2019.09.020.

Fishburn, J., E. Tomko, E. Galburt, and S. Hahn. 2015. Double-stranded DNA translocase activity of transcription factor TFIIH and the mechanism of RNA polymerase II open complex formation. *Proc. Natl. Acad. Sci. U.S.A.* 112:3961–3966. doi:10.1073/pnas.1417709112.

Fitz-James, M.H., P. Tong, A.L. Pidoux, H. Ozadam, L. Yang, S.A. White, J. Dekker, and R.C. Allshire. 2020. Large domains of heterochromatin direct the formation of short mitotic chromosome loops. *Elife*. 9. doi:10.7554/eLife.57212.

Fregoso, M., J.-P. Lainé, J. Aguilar-Fuentes, V. Mocquet, E. Reynaud, F. Coin, J.-M. Egly, and M. Zurita. 2007. DNA Repair and Transcriptional Deficiencies Caused by Mutations in the Drosophila p52 Subunit of TFIIH Generate Developmental Defects and Chromosome Fragility. *Mol Cell Biol*. 27:3640–3650. doi:10.1128/mcb.00030-07.

Ganji, M., I.A. Shaltiel, S. Bisht, E. Kim, A. Kalichava, C.H. Haering, and C. Dekker. 2018. Real-time imaging of DNA loop extrusion by condensin. *Science*. 360:102–105. doi:10.1126/science.aar7831.

Garcia-Luis, J., L. Lazar-Stefanita, P. Gutierrez-Escribano, A. Thierry, A. Cournac, A. García, S. González, M. Sánchez, A. Jarmuz, A. Montoya, M. Dore, H. Kramer, M.M. Karimi, F. Antequera, R. Koszul, and L. Aragón. 2019. FACT mediates cohesin function on chromatin. *Nat. Struct. Mol. Biol.* 26:970–979. doi:10.1038/s41594-019-0307-x.

Gibcus, J.H., K. Samejima, A. Goloborodko, I. Samejima, N. Naumova, J. Nuebler, M.T. Kanemaki, L. Xie, J.R. Paulson, W.C. Earnshaw, L.A. Mirny, and J. Dekker. 2018. A pathway for mitotic chromosome formation. *Science*. 359:eaao6135. doi:10.1126/science.aao6135.

Gilchrist, D.A., G. Dos Santos, D.C. Fargo, B. Xie, Y. Gao, L. Li, and K. Adelman. 2010. Pausing of RNA polymerase II disrupts DNA-specified nucleosome organization to enable precise gene regulation. *Cell*. 143:540–551. doi:10.1016/j.cell.2010.10.004.

Glover-Cutter, K., S. Larochelle, B. Erickson, C. Zhang, K. Shokat, R.P. Fisher, and D.L. Bentley. 2009. TFIIH-associated Cdk7 kinase functions in phosphorylation of C-terminal domain Ser7 residues, promoter-proximal pausing, and termination by RNA polymerase II. *Mol. Cell. Biol.* 29:5455–5464. doi:10.1128/MCB.00637-09.

Goloborodko, A., J.F. Marko, and L.A. Mirny. 2016a. Chromosome Compaction by Active Loop Extrusion. *Biophys J*. 110:2162–2168. doi:10.1016/j.bpj.2016.02.041.

Goloborodko, A., M.V. Imakaev, J.F. Marko, and L. Mirny. 2016b. Compaction and segregation of sister chromatids via active loop extrusion. *Elife*. 5. doi:10.7554/eLife.14864.

Green, L.C., P. Kalitsis, T.M. Chang, M. Cipetic, J.H. Kim, O. Marshall, L. Turnbull, C.B. Whitchurch, P. Vagnarelli, K. Samejima, W.C. Earnshaw, K.H.A. Choo, and D.F. Hudson. 2012. Contrasting roles of condensin I and condensin II in mitotic chromosome formation. *J. Cell. Sci.* 125:1591–1604. doi:10.1242/jcs.097790.

Grenfell, A.W., R. Heald, and M. Strzelecka. 2016. Mitotic noncoding RNA processing promotes kinetochore and spindle assembly in Xenopus. *J. Cell Biol.* 214:133–141. doi:10.1083/jcb.201604029.

Grigoryev, S.A., G. Bascom, J.M. Buckwalter, M.B. Schubert, C.L. Woodcock, and T. Schlick. 2016. Hierarchical looping of zigzag nucleosome chains in metaphase chromosomes. *Proc. Natl. Acad. Sci. U.S.A.* 113:1238–1243. doi:10.1073/pnas.1518280113.

Groen, A.C., M. Coughlin, and T.J. Mitchison. 2011. Microtubule assembly in meiotic extract requires glycogen. *Mol. Biol. Cell*. 22:3139–3151. doi:10.1091/mbc.E11-02-0158.

Grünberg, S., L. Warfield, and S. Hahn. 2012. Architecture of the RNA polymerase II preinitiation complex and mechanism of ATP-dependent promoter opening. *Nat. Struct. Mol. Biol.* 19:788–796. doi:10.1038/nsmb.2334.

Haase, J., M.K. Bonner, H. Halas, and A.E. Kelly. 2017. Distinct Roles of the Chromosomal Passenger Complex in the Detection of and Response to Errors in Kinetochore-Microtubule Attachment. *Dev. Cell*. 42:640–654.e5. doi:10.1016/j.devcel.2017.08.022.

Hayashi-Takanaka, Y., K. Maehara, A. Harada, T. Umehara, S. Yokoyama, C. Obuse, Y. Ohkawa, N. Nozaki, and H. Kimura. 2015. Distribution of histone H4 modifications as revealed by a panel of specific monoclonal antibodies. *Chromosome Res.* 23:753–766. doi:10.1007/s10577-015-9486-4.

Herrera-Cruz, M., G. Cruz, V. Valadez-Graham, M. Fregoso-Lomas, C. Villicaña, M. Vázquez, E. Reynaud, and M. Zurita. 2012. Physical and functional interactions between Drosophila homologue of Swc6/p18Hamlet subunit of the SWR1/SRCAP chromatin-remodeling complex with the DNA repair/transcription factor TFIIH. *J. Biol. Chem.* 287:33567–33580. doi:10.1074/jbc.m112.383505.

Hirano, T. 2014. Condensins and the evolution of torsion-mediated genome organization. *Trends Cell Biol.* 24:727–733. doi:10.1016/j.tcb.2014.06.007.

Hirano, T., and T.J. Mitchison. 1993. Topoisomerase II does not play a scaffolding role in the organization of mitotic chromosomes assembled in Xenopus egg extracts. *J. Cell Biol.* 120:601–612.

Hirano, T., and T.J. Mitchison. 1994. A heterodimeric coiled-coil protein required for mitotic chromosome condensation in vitro. *Cell*. 79:449–458. doi:10.1016/0092-8674(94)90254-2.

Hirano, T., R. Kobayashi, and M. Hirano. 1997. Condensins, chromosome condensation protein complexes containing XCAP-C, XCAP-E and a Xenopus homolog of the Drosophila Barren protein. *Cell*. 89:511–521.

Hirota, T., D. Gerlich, B. Koch, J. Ellenberg, and J.-M. Peters. 2004. Distinct functions of condensin I and II in mitotic chromosome assembly. *J. Cell. Sci.* 117:6435–6445. doi:10.1242/jcs.01604.

Hoffman, D.B., C.G. Pearson, T.J. Yen, B.J. Howell, and E.D. Salmon. 2001. Microtubule-dependent changes in assembly of microtubule motor proteins and mitotic spindle checkpoint proteins at PtK1 kinetochores. *Mol. Biol. Cell*. 12:1995–2009.

Ito, S., I. Kuraoka, P. Chymkowitch, E. Compe, A. Takedachi, C. Ishigami, F. Coin, J.-M. Egly, and K. Tanaka. 2007. XPG stabilizes TFIIH, allowing transactivation of nuclear receptors: implications for Cockayne syndrome in XP-G/CS patients. *Mol. Cell*. 26:231–243. doi:10.1016/j.molcel.2007.03.013.

Jukam, D., S.A.M. Shariati, and J.M. Skotheim. 2017. Zygotic Genome Activation in Vertebrates. *Dev. Cell*. 42:316–332. doi:10.1016/j.devcel.2017.07.026.

Kappenberger, J., W. Koelmel, E. Schoenwetter, T. Scheuer, J. Woerner, J. Kuper, and C. Kisker. 2020. How to limit the speed of a motor: the intricate regulation of the XPB ATPase and translocase in TFIIH. *Nucleic Acids Res.* 48:12282–12296. doi:10.1093/nar/gkaa911.

Ke, Y., Y. Xu, X. Chen, S. Feng, Z. Liu, Y. Sun, X. Yao, F. Li, W. Zhu, L. Gao, H. Chen, Z. Du, W. Xie, X. Xu, X. Huang, and J. Liu. 2017. 3D Chromatin Structures of Mature Gametes and Structural Reprogramming during Mammalian Embryogenesis. *Cell*. 170:367–381.e20. doi:10.1016/j.cell.2017.06.029.

Kelly, A.E., C. Ghenoiu, J.Z. Xue, C. Zierhut, H. Kimura, and H. Funabiki. 2010. Survivin reads phosphorylated histone H3 threonine 3 to activate the mitotic kinase Aurora B. *Science*. 330:235–239. doi:10.1126/science.1189505.

Kim, E., A.M. Gonzalez, B. Pradhan, J. van der Torre, and C. Dekker. 2021. Condensin-driven loop extrusion on supercoiled DNA. *bioRxiv*. 2021.05.15.444164. doi:10.1101/2021.05.15.444164.

Kim, J.H., T. Zhang, N.C. Wong, N. Davidson, J. Maksimovic, A. Oshlack, W.C. Earnshaw, P. Kalitsis, and D.F. Hudson. 2013. Condensin I associates with structural and gene regulatory regions in vertebrate chromosomes. *Nat Commun*. 4:2537. doi:10.1038/ncomms3537.

Kim, K.-D., H. Tanizawa, O. Iwasaki, and K.-I. Noma. 2016. Transcription factors mediate condensin recruitment and global chromosomal organization in fission yeast. *Nat. Genet.* 48:1242–1252. doi:10.1038/ng.3647.

Kim, T.K., R.H. Ebright, and D. Reinberg. 2000. Mechanism of ATP-dependent promoter melting by transcription factor IIH. *Science*. 288:1418–1422. doi:10.1126/science.288.5470.1418.

Kimura, K., M. Hirano, R. Kobayashi, and T. Hirano. 1998. Phosphorylation and activation of 13S condensin by Cdc2 in vitro. *Science*. 282:487–490. doi:10.1126/science.282.5388.487.

Kinoshita, K., and T. Hirano. 2017. Dynamic organization of mitotic chromosomes. *Curr. Opin. Cell Biol.* 46:46–53. doi:10.1016/j.ceb.2017.01.006.

Kinoshita, K., T.J. Kobayashi, and T. Hirano. 2015. Balancing acts of two HEAT subunits of condensin I support dynamic assembly of chromosome axes. *Dev. Cell*. 33:94–106. doi:10.1016/j.devcel.2015.01.034.

Klein Douwel, D., R.A.C.M. Boonen, D.T. Long, A.A. Szypowska, M. Räschle, J.C. Walter, and P. Knipscheer. 2014. XPF-ERCC1 acts in Unhooking DNA interstrand crosslinks in cooperation with FANCD2 and FANCP/SLX4. *Mol. Cell*. 54:460–471. doi:10.1016/j.molcel.2014.03.015.

Klein Douwel, D., W.S. Hoogenboom, R.A. Boonen, and P. Knipscheer. 2017. Recruitment and positioning determine the specific role of the XPF-ERCC1 endonuclease in interstrand crosslink repair. *EMBO J.* 36:2034–2046. doi:10.15252/embj.201695223.

Kolesnikova, O., L. Radu, and A. Poterszman. 2019. TFIIH: A multi-subunit complex at the cross-roads of transcription and DNA repair. *Adv Protein Chem Struct Biol*. 115:21–67. doi:10.1016/bs.apcsb.2019.01.003.

Kong, M., E.E. Cutts, D. Pan, F. Beuron, T. Kaliyappan, C. Xue, E.P. Morris, A. Musacchio, A. Vannini, and E.C. Greene. 2020. Human Condensin I and II Drive Extensive ATP-Dependent Compaction of Nucleosome-Bound DNA. *Mol. Cell*. 79:99–114.e9. doi:10.1016/j.molcel.2020.04.026.

Kranz, A.-L., C.-Y. Jiao, L.H. Winterkorn, S.E. Albritton, M. Kramer, and S. Ercan. 2013. Genome-wide analysis of condensin binding in Caenorhabditis elegans. *Genome Biol.* 14:R112–15. doi:10.1186/gb-2013-14-10-r112.

Kwiatkowski, N., T. Zhang, P.B. Rahl, B.J. Abraham, J. Reddy, S.B. Ficarro, A. Dastur, A. Amzallag, S. Ramaswamy, B. Tesar, C.E. Jenkins, N.M. Hannett, D. McMillin, T. Sanda, T. Sim, N.D. Kim, T. Look, C.S. Mitsiades, A.P. Weng, J.R. Brown, C.H. Benes, J.A. Marto, R.A. Young, and N.S. Gray. 2014. Targeting transcription regulation in cancer with a covalent CDK7 inhibitor. *Nature*. 511:616–620. doi:10.1038/nature13393.

Larochelle, S., K.A. Merrick, M.-E. Terret, L. Wohlbold, N.M. Barboza, C. Zhang, K.M. Shokat, P.V. Jallepalli, and R.P. Fisher. 2007. Requirements for Cdk7 in the assembly of Cdk1/cyclin B and activation of Cdk2 revealed by chemical genetics in human cells. *Mol. Cell*. 25:839–850. doi:10.1016/j.molcel.2007.02.003.

Liu, W., B. Tanasa, O.V. Tyurina, T.Y. Zhou, R. Gassmann, W.T. Liu, K.A. Ohgi, C. Benner, I. Garcia-Bassets, A.K. Aggarwal, A. Desai, P.C. Dorrestein, C.K. Glass, and M.G. Rosenfeld. 2010. PHF8 mediates histone H4 lysine 20 demethylation events involved in cell cycle progression. *Nature*. 466:508–512. doi:10.1038/nature09272.

Maddox, P.S., N. Portier, A. Desai, and K. Oegema. 2006. Molecular analysis of mitotic chromosome condensation using a quantitative time-resolved fluorescence microscopy assay. *Proc. Natl. Acad. Sci. U.S.A.* 103:15097–15102. doi:10.1073/pnas.0606993103.

Maeshima, K., and U.K. Laemmli. 2003. A two-step scaffolding model for mitotic chromosome assembly. *Dev. Cell*. 4:467–480. doi:10.1016/s1534-5807(03)00092-3.

Maresca, T.J., and R. Heald. 2006. Methods for studying spindle assembly and chromosome condensation in Xenopus egg extracts. *Methods Mol. Biol.* 322:459–474. doi:10.1007/978-1-59745-000-3_33.

Mizuki, F., T. Namiki, H. Sato, H. Furukawa, T. Matsusaka, Y. Ohshima, R. Ishibashi, T. Andoh, and T. Tani. 2007. Participation of XPB/Ptr8p, a component of TFIIH, in nucleocytoplasmic transport of mRNA in fission yeast. *Genes Cells*. 12:35–47. doi:10.1111/j.1365-2443.2006.01032.x.

Nakazawa, N., K. Sajiki, X. Xu, A. Villar-Briones, O. Arakawa, and M. Yanagida. 2015. RNA pol II transcript abundance controls condensin accumulation at mitotically up-regulated and heat-shock-inducible genes in fission yeast. *Genes Cells*. 20:481–499. doi:10.1111/gtc.12239.

Nakazawa, N., R. Mehrotra, M. Ebe, and M. Yanagida. 2011. Condensin phosphorylated by the Aurora-B-like kinase Ark1 is continuously required until telophase in a mode distinct from Top2. *J. Cell. Sci.* 124:1795–1807. doi:10.1242/jcs.078733.

Naumova, N., M. Imakaev, G. Fudenberg, Y. Zhan, B.R. Lajoie, L.A. Mirny, and J. Dekker. 2013. Organization of the mitotic chromosome. *Science*. 342:948–953. doi:10.1126/science.1236083.

Nielsen, C.F., T. Zhang, M. Barisic, P. Kalitsis, and D.F. Hudson. 2020. Topoisomerase IIα is essential for maintenance of mitotic chromosome structure. *Proc. Natl. Acad. Sci. U.S.A.* 117:12131–12142. doi:10.1073/pnas.2001760117.

Nogales, E., and B.J. Greber. 2019. High-resolution cryo-EM structures of TFIIH and their functional implications. *Curr. Opin. Struct. Biol.* 59:188–194. doi:10.1016/j.sbi.2019.08.002.

Ono, T., A. Losada, M. Hirano, M.P. Myers, A.F. Neuwald, and T. Hirano. 2003. Differential contributions of condensin I and condensin II to mitotic chromosome architecture in vertebrate cells. *Cell*. 115:109–121. doi:10.1016/s0092-8674(03)00724-4.

Ono, T., Y. Fang, D.L. Spector, and T. Hirano. 2004. Spatial and temporal regulation of Condensins I and II in mitotic chromosome assembly in human cells. *Mol. Biol. Cell*. 15:3296–3308. doi:10.1091/mbc.e04-03-0242.

Ou, H.D., S. Phan, T.J. Deerinck, A. Thor, M.H. Ellisman, and C.C. O'Shea. 2017. ChromEMT: Visualizing 3D chromatin structure and compaction in interphase and mitotic cells. *Science*. 357. doi:10.1126/science.aag0025.

Palozola, K.C., J. Lerner, and K.S. Zaret. 2019. A changing paradigm of transcriptional memory propagation through mitosis. *Nat. Rev. Mol. Cell Biol.* 20:55–64. doi:10.1038/s41580-018-0077-z.

Pandey, N., D. Keifenheim, M.M. Yoshida, V.A. Hassebroek, C. Soroka, Y. Azuma, and D.J. Clarke. 2020. Topoisomerase II SUMOylation activates a metaphase checkpoint via Haspin and Aurora B kinases. *J. Cell Biol.* 219. doi:10.1083/jcb.201807189.

Paulson, J.R., and U.K. Laemmli. 1977. The structure of histone-depleted metaphase chromosomes. *Cell*. 12:817–828. doi:10.1016/0092-8674(77)90280-x.

Paulson, J.R., D.F. Hudson, F. Cisneros-Soberanis, and W.C. Earnshaw. 2021. Mitotic chromosomes. *Semin Cell Dev Biol*. 117:7–29. doi:10.1016/j.semcdb.2021.03.014.

Perea-Resa, C., L. Bury, I.M. Cheeseman, and M.D. Blower. 2020. Cohesin Removal Reprograms Gene Expression upon Mitotic Entry. *Mol. Cell*. doi:10.1016/j.molcel.2020.01.023.

Petrenko, N., Y. Jin, L. Dong, K.H. Wong, and K. Struhl. 2019. Requirements for RNA polymerase II preinitiation complex formation in vivo. *Elife*. 8:699. doi:10.7554/eLife.43654.

Piazza, I., A. Rutkowska, A. Ori, M. Walczak, J. Metz, V. Pelechano, M. Beck, and C.H. Haering. 2014. Association of condensin with chromosomes depends on DNA binding by its HEAT-repeat subunits. *Nat. Struct. Mol. Biol.* 21:560–568. doi:10.1038/nsmb.2831.

Piskadlo, E., A. Tavares, and R.A. Oliveira. 2017. Metaphase chromosome structure is dynamically maintained by condensin I-directed DNA (de)catenation. *Elife*. 6. doi:10.7554/eLife.26120.

Pradhan, B., R. Barth, E. Kim, I.F. Davidson, B. Bauer, T. van Laar, W. Yang, J.-K. Ryu, J. van der Torre, J.-M. Peters, and C. Dekker. 2021. SMC complexes can traverse physical roadblocks bigger than their ring size. *bioRxiv*. 2021.07.15.452501. doi:10.1101/2021.07.15.452501.

Presler, M., E. Van Itallie, A.M. Klein, R. Kunz, M.L. Coughlin, L. Peshkin, S.P. Gygi, M. Wühr, and M.W. Kirschner. 2017. Proteomics of phosphorylation and protein dynamics during fertilization and meiotic exit in the Xenopus egg. *Proc. Natl. Acad. Sci. U.S.A.* 114:E10838–E10847. doi:10.1073/pnas.1709207114.

Rimel, J.K., and D.J. Taatjes. 2018. The essential and multifunctional TFIIH complex. *Protein Sci.* 27:1018–1037. doi:10.1002/pro.3424.

Rivosecchi, J., L. Vachez, F. Gautier, P. Bernard, and V. Vanoosthuyse. 2020. RNA polymerase backtracking drives the accumulation of fission yeast condensin at active genes. *bioRxiv*. 9:2020.11.13.381434. doi:10.1101/2020.11.13.381434.

Robellet, X., V. Vanoosthuyse, and P. Bernard. 2017. The loading of condensin in the context of chromatin. *Curr. Genet.* 63:577–589. doi:10.1007/s00294-016-0669-0.

Sakai, A., K. Hizume, T. Sutani, K. Takeyasu, and M. Yanagida. 2003. Condensin but not cohesin SMC heterodimer induces DNA reannealing through protein-protein assembly. *EMBO J.* 22:2764–2775. doi:10.1093/emboj/cdg247.

Samejima, K., D.G. Booth, H. Ogawa, J.R. Paulson, L. Xie, C.A. Watson, M. Platani, M.T. Kanemaki, and W.C. Earnshaw. 2018. Functional analysis after rapid degradation of condensins and 3D-EM reveals chromatin volume is uncoupled from chromosome architecture in mitosis. *J. Cell. Sci.* 131. doi:10.1242/jcs.210187.

Samejima, K., I. Samejima, P. Vagnarelli, H. Ogawa, G. Vargiu, D.A. Kelly, F. de Lima Alves, A. Kerr, L.C. Green, D.F. Hudson, S. Ohta, C.A. Cooke, C.J. Farr, J. Rappsilber, and W.C. Earnshaw. 2012. Mitotic chromosomes are compacted laterally by KIF4 and condensin and axially by topoisomerase IIα. *J. Cell Biol.* 199:755–770. doi:10.1083/jcb.201202155.

Sandoz, J., Z. Nagy, P. Catez, G. Caliskan, S. Geny, J.-B. Renaud, J.-P. Concordet, A. Poterszman, L. Tora, J.-M. Egly, N.L. May, and F. Coin. 2019. Functional interplay between TFIIH and KAT2A regulates higher-order chromatin structure and class II gene expression. *Nat Commun*. 10:1288. doi:10.1038/s41467-019-09270-2.

Schilbach, S., S. Aibara, C. Dienemann, F. Grabbe, and P. Cramer. 2021. Structure of RNA polymerase II pre-initiation complex at 2.9 Å defines initial DNA opening. *Cell*. 184:4064–4072.e28. doi:10.1016/j.cell.2021.05.012.

Schmidt, C.K., N. Brookes, and F. Uhlmann. 2009. Conserved features of cohesin binding along fission yeast chromosomes. *Genome Biol.* 10:R52–16. doi:10.1186/gb-2009-10-5-r52.

Shechter, D., R.K. Chitta, A. Xiao, J. Shabanowitz, D.F. Hunt, and C.D. Allis. 2009. A distinct H2A.X isoform is enriched in Xenopus laevis eggs and early embryos and is phosphorylated in the absence of a checkpoint. *Proc. Natl. Acad. Sci. U.S.A.* 106:749–754. doi:10.1073/pnas.0812207106.

Shevchenko, A., H. Tomas, J. Havlis, J.V. Olsen, and M. Mann. 2006. In-gel digestion for mass spectrometric characterization of proteins and proteomes. *Nat Protoc*. 1:2856–2860. doi:10.1038/nprot.2006.468.

Shintomi, K., and T. Hirano. 2011. The relative ratio of condensin I to II determines chromosome shapes. *Genes Dev.* 25:1464–1469. doi:10.1101/gad.2060311.

Shintomi, K., and T. Hirano. 2021. Guiding functions of the C-terminal domain of topoisomerase IIα advance mitotic chromosome assembly. *Nat Commun*. 12:2917–13. doi:10.1038/s41467-021-23205-w.

Shintomi, K., F. Inoue, H. Watanabe, K. Ohsumi, M. Ohsugi, and T. Hirano. 2017. Mitotic chromosome assembly despite nucleosome depletion in Xenopus egg extracts. *Science*. 17:eaam9702–1287. doi:10.1126/science.aam9702.

Shintomi, K., T.S. Takahashi, and T. Hirano. 2015. Reconstitution of mitotic chromatids with a minimum set of purified factors. *Nat. Cell Biol.* 17:1014–1023. doi:10.1038/ncb3187.

Sutani, T., T. Sakata, R. Nakato, K. Masuda, M. Ishibashi, D. Yamashita, Y. Suzuki, T. Hirano, M. Bando, and K. Shirahige. 2015. Condensin targets and reduces unwound DNA structures associated with transcription in mitotic chromosome condensation. *Nat Commun*. 6:7815. doi:10.1038/ncomms8815.

Tada, K., H. Susumu, T. Sakuno, and Y. Watanabe. 2011. Condensin association with histone H2A shapes mitotic chromosomes. *Nature*. 474:477–483. doi:10.1038/nature10179.

Thadani, R., J. Kamenz, S. Heeger, S. Muñoz, and F. Uhlmann. 2018. Cell-Cycle Regulation of Dynamic Chromosome Association of the Condensin Complex. *Cell Rep*. 23:2308–2317. doi:10.1016/j.celrep.2018.04.082.

Thakur, J., and S. Henikoff. 2020. Architectural RNA in chromatin organization. *Biochem. Soc. Trans.* 48:1967–1978. doi:10.1042/BST20191226.

Titov, D.V., B. Gilman, Q.-L. He, S. Bhat, W.-K. Low, Y. Dang, M. Smeaton, A.L. Demain, P.S. Miller, J.F. Kugel, J.A. Goodrich, and J.O. Liu. 2011. XPB, a subunit of TFIIH, is a target of the natural product triptolide. *Nat. Chem. Biol.* 7:182–188. doi:10.1038/nchembio.522.

Tomko, E.J., J. Fishburn, S. Hahn, and E.A. Galburt. 2017. TFIIH generates a six-base-pair open complex during RNAP II transcription initiation and start-site scanning. *Nat. Struct. Mol. Biol.* 24:1139–1145. doi:10.1038/nsmb.3500.

Toselli-Mollereau, E., X. Robellet, L. Fauque, S. Lemaire, C. Schiklenk, C. Klein, C. Hocquet, P. Legros, L. N'Guyen, L. Mouillard, E. Chautard, D. Auboeuf, C.H. Haering, and P. Bernard. 2016. Nucleosome eviction in mitosis assists condensin loading and chromosome condensation. *EMBO J.* 35:1565–1581. doi:10.15252/embj.201592849.

Vasquez, P.A., C. Hult, D. Adalsteinsson, J. Lawrimore, M.G. Forest, and K. Bloom. 2016. Entropy gives rise to topologically associating domains. *Nucleic Acids Res.* 44:5540–5549. doi:10.1093/nar/gkw510.

Walther, N., M.J. Hossain, A.Z. Politi, B. Koch, M. Kueblbeck, Ø. Ødegård-Fougner, M. Lampe, and J. Ellenberg. 2018. A quantitative map of human Condensins provides new insights into mitotic chromosome architecture. *J. Cell Biol.* 217:2309–2328. doi:10.1083/jcb.201801048.

Wang, W.-L., L.C. Anderson, J.J. Nicklay, H. Chen, M.J. Gamble, J. Shabanowitz, D.F. Hunt, and D. Shechter. 2014. Phosphorylation and arginine methylation mark histone H2A prior to deposition during Xenopus laevis development. *Epigenetics Chromatin*. 7:22–20. doi:10.1186/1756-8935-7-22.

Wühr, M., R.M. Freeman, M. Presler, M.E. Horb, L. Peshkin, S.P. Gygi, and M.W. Kirschner. 2014. Deep proteomics of the Xenopus laevis egg using an mRNA-derived reference database. *Curr. Biol.* 24:1467–1475. doi:10.1016/j.cub.2014.05.044.

Zentner, G.E., and S. Henikoff. 2013. Regulation of nucleosome dynamics by histone modifications. *Nat Struct Mol Biol*. 20:259–266. doi:10.1038/nsmb.2470.

Zhou, C.Y., and R. Heald. 2020. Emergent properties of mitotic chromosomes. *Curr. Opin. Cell Biol.* 64:43–49. doi:10.1016/j.ceb.2020.02.003.

Zhou, K., Y. Liu, and K. Luger. 2020. Histone chaperone FACT FAcilitates Chromatin Transcription: mechanistic and structural insights. *Curr. Opin. Struct. Biol.* 65:26–32. doi:10.1016/j.sbi.2020.05.019.

Zierhut, C., C. Jenness, H. Kimura, and H. Funabiki. 2014. Nucleosomal regulation of chromatin composition and nuclear assembly revealed by histone depletion. *Nat. Struct. Mol. Biol.* 21:617–625. doi:10.1038/nsmb.2845.
